# Frequent Genetic Mismatch between Vaccine Strains and Circulating Seasonal Influenza Viruses, Hong Kong, China, 1996–2012

**DOI:** 10.3201/eid2410.180652

**Published:** 2018-10

**Authors:** Martin C.W. Chan, Maggie H. Wang, Zigui Chen, David S.C. Hui, Angela K. Kwok, Apple C.M. Yeung, Kun M. Liu, Yun Kit Yeoh, Nelson Lee, Paul K.S. Chan

**Affiliations:** Author affiliation: The Chinese University of Hong Kong, Hong Kong, China

**Keywords:** Molecular epidemiology, hemagglutinin, genetic drift, antigenic drift, subtropics, China, Hong Kong, viruses, influenza, vaccines, vaccination

## Abstract

The World Health Organization selects influenza vaccine compositions biannually to cater to peaks in temperate regions. In tropical and subtropical regions, where influenza seasonality varies and epidemics can occur year-round, the choice of vaccine remains uncertain. Our 17-year molecular epidemiologic survey showed that most influenza A(H3N2) (9/11) and B (6/7) vaccine strains had circulated in East Asia >1 year before inclusion into vaccines. Northern Hemisphere vaccine strains and circulating strains in East Asia were closely matched in 7 (20.6%) of 34 seasons for H3N2 and 5 (14.7%) of 34 seasons for B. Southern Hemisphere vaccines also had a low probability of matching (H3N2, 14.7%; B, 11.1%). Strain drift among seasons was common (H3N2, 41.2%; B, 35.3%), and biannual vaccination strategy (Northern Hemisphere vaccines in November followed by Southern Hemisphere vaccines in May) did not improve matching. East Asia is an important contributor to influenza surveillance but often has mismatch between vaccine and contemporarily circulating strains.

Influenza is a major cause of illness and death worldwide. The human influenza virus spreads rapidly (average reproduction number of 1.28), typically infects 5%–10% of the population during seasonal epidemics, and results in 3–5 million cases of severe acute lower respiratory tract infection and ≈500,000 deaths per year globally (*1*,*2*). Continuous evolution of the single-stranded influenza virus results in antigenic drift of its surface proteins hemagglutinin (HA) and neuraminidase (*3*,*4*). Antigenic drift occurs on average 2–8 years in response to host immune selection pressure and is most frequently seen in influenza A(H3N2), followed by influenza B and influenza A(H1N1). Antigenic drift confers the virus an ability to escape immunity induced by previous infection or immunization (*5*).

Effectiveness of current influenza vaccines is predominantly determined by matching between vaccines and circulating strains (*6*). To achieve the best possible vaccine matching, the World Health Organization (WHO) has changed its recommended vaccine composition 12 times for influenza A(H3N2), 10 times for influenza B, and 6 times for influenza A(H1N1) since 1998 (*7*). WHO reviews influenza vaccine composition each February and September to provide timely recommendation for temperate regions of the Northern and Southern Hemispheres, respectively. Tropical and subtropical regions are expected to choose 1 of the 2 recommended vaccine compositions. However, influenza seasonality varies more in the tropics and subtropics, where epidemics can occur year-round. Moreover, a national policy on the choice and timing of vaccination is lacking in many tropical and subtropical countries, where 60% of the world’s population resides (*8*).

We analyzed the 2 most frequently drifting influenza types, A(H3N2) and B, in Hong Kong during 1996–2012 to examine matching between vaccine strains and circulating field viruses to document the challenges faced in this region. We evaluated options on choosing the Northern or Southern Hemisphere vaccine compositions and the strategy of an annual or biannual vaccination.

## Methods

### Study Samples

We conducted a retrospective molecular epidemiologic study to analyze the HA sequences of influenza A(H3N2) and influenza B viruses that circulated during a 17-year period (1996–2012) in Hong Kong, a subtropical city in East Asia (22°17′7.87′′N, 114°9′27.68′′E) with a high population density of 57,250 persons/km^2^ in the most densely populated district, Kwun Tong (*9*). Hong Kong is an international hub. As recorded in 2011, Hong Kong received >34 million visitors from neighboring regions, including South and Southeast Asia, Taiwan, mainland China, and Macao, and an average of ≈650,000 cross-boundary trips between Hong Kong and mainland China were recorded daily (*10*,*11*). In this study, we used Hong Kong as a proxy to reflect the challenges faced in East Asia.

We focused on influenza A(H3N2) and influenza B, rather than A(H1N1), because changes in vaccine composition were more frequent for the former 2 viruses. Clinical specimens were derived from patients admitted to the Prince of Wales Hospital, which serves 9% (0.6 million) of Hong Kong’s population (*12*). We sequenced an average of 30 influenza A(H3N2) and 30 influenza B clinical samples per year, except for 2009, when influenza A(H1N1)pdm09 predominated. Selection was prioritized for samples collected during different months to reveal the whole annual spectrum of circulating viruses.

### Identifying Closest Vaccine Strains and Matched Seasons

We identified vaccine strain closest to each circulating virus on the basis of HA sequence analysis. We extracted viral RNA from specimens using the PureLink Viral RNA/DNA Mini kit (Thermo Fisher, Waltham, MA, USA). The full-length influenza A(H3N2) HA encoding region was amplified and Sanger sequenced according to our previously described method (*13*). Similarly, the full-length influenza B HA encoding region was amplified with primers FLUB-UNI9-HAF1 (5′-AGC AGA AGC AGA GCA TTT TYT AAT ATC-3′) and FLUB-HAR1 (5′-ACA AGC AAA CAA GYA CYA CAA YAA AG-3′) and Sanger sequenced. We downloaded ancestral influenza A(H3N2) and influenza B HA sequences from GenBank. Information about WHO-recommended vaccine strains and sequences were retrieved from the Influenza Research Database (*14*). When the recommendation included alternative vaccine strains, each of them was included for analysis.

We conducted neighbor-joining phylogenetic inference on the basis of the HA sequences using MEGA6 (*15*). Resulting trees were visualized using FigTree version 1.4.2. We then matched each circulating strain to a closest influenza A(H3N2) or influenza B vaccine strain based on pairwise protein sequence distances as calculated by the Poisson model (*16*). Circulating strains with equal distance to 2 vaccine strains were assigned to the vaccine recommended in an earlier year. Strains with distances >2 times the SD from the mean distance of all strains were regarded as outliers and thus had no closest vaccine strain assigned.

When the circulating viruses and their closest vaccine strain corresponded in time, the season was regarded as matched. On the other hand, when the circulating viruses coincided with vaccine strains from a previous season (e.g., circulating strain from 2012 matching vaccine strain from 2010), the season was regarded as not matched. This matching scheme was based on the rationale that WHO changes the influenza vaccine composition only when substantial antigenic drift has occurred, and suboptimal effectiveness would be expected for the previous vaccine compositions.

### Seasonality and Protection Period

Unlike temperate regions, peak seasons of influenza in the subtropics are not restricted to winter months. Because previous studies have revealed a biannual pattern of influenza activity in Hong Kong and surrounding regions (*12*,*17*), we divided the surveyed 17-year period into 34 influenza seasons. We grouped calendar months under winter and summer influenza seasons on the basis of the average monthly number of hospital admissions of patients for whom influenza was laboratory confirmed that were recorded during the study period.

The Hong Kong Government Influenza Vaccination Programme recommends use of the Northern Hemisphere influenza vaccines and begins annual vaccination every November. Therefore, we defined the protection period of Northern Hemisphere vaccines as from November through October of the following year. Although Southern Hemisphere vaccines are not typically used in Hong Kong, for this analysis we regarded the protection period of these vaccines to be from May through April of the following year.

## Results

**Figure 1:**
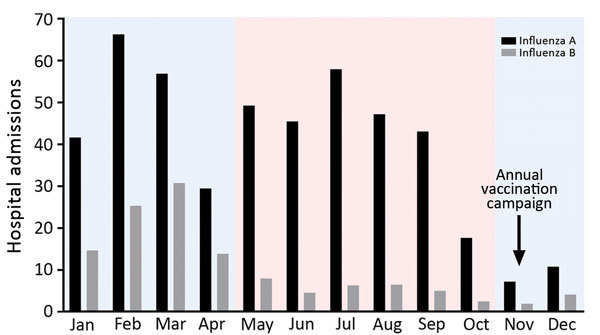
Seasonality of influenza A and B, Hong Kong, China, 1996–2012. The numbers of patients hospitalized with acute respiratory illnesses and who had laboratory-confirmed influenza were retrieved from a computerized laboratory information system at the microbiology department of a district general hospital that serves 9% (0.6 million) of Hong Kong’s population. Pink indicates summer season; blue indicates winter season.

During the 17-year study period, 8,011 persons with influenza A and 2,079 with influenza B were admitted to the Prince of Wales Hospital. Both infections exhibited 2 seasonal peaks in most years (Figure 1). Most influenza admissions occurred from late winter through early spring (late January through early March) and around summer (May–September), although the summer peak of influenza B varied more and was less prominent than that of influenza A. Based on this observed seasonality, we grouped circulating strains collected during November–April under the winter season and those collected during May–October under the summer season.

**Figure 2:**
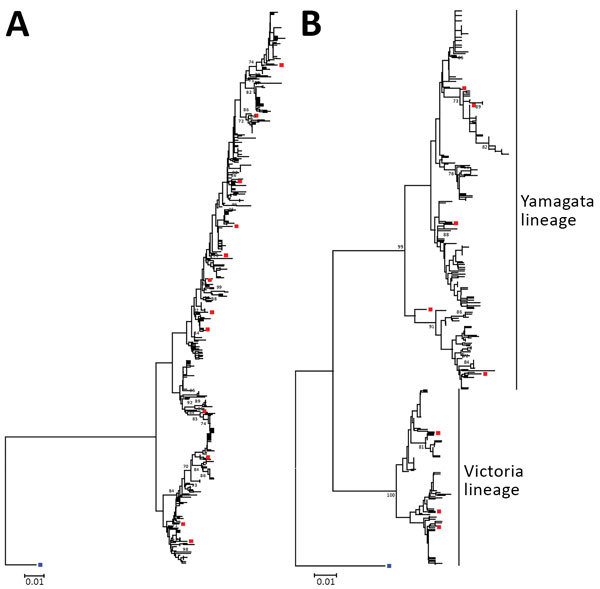
Neighbor-joining phylogenetic inference of near-complete hemagglutinin protein of influenza A(H3N2) (A) and influenza B (B), Hong Kong, China, 1996–2012. Pairwise protein sequence distances were calculated using a Poisson model. Blue squares denote ancestral influenza strains: influenza A(H3N2), A/Hong Kong/1/1968, GenBank accession no. CY044261; influenza B, B/Lee/1940, accession no. CY115111. Red squares denote recommended vaccine strains; for clarity, only those used in Figures 3 and 4 are shown. Bootstrap values >70% are shown at the respective nodes. All alignment positions containing gaps were omitted from the analysis. Trees were rooted to ancestral strains and drawn to scale. Scale bars indicate numbers of amino acid substitutions per position. GenBank accession numbers of hemagglutinin sequences of circulating strains collected in this study are shown in the Technical Appendix Table.

We successfully sequenced 502 influenza A(H3N2) and 481 influenza B samples covering all influenza seasons in the study period, except in 2009, when influenza A(H1N1)pdm09 dominated activity. In the phylogenetic analysis based on HA sequences, H3N2 virus exhibited a characteristic ladder-like tree topology suggesting continuous unidirectional evolution (Figure 2, panel A). Existing H3N2 variants circulated for a median of 2 years (range 1–4 years), with gaps between the winter and summer seasons, and became displaced rapidly by newly emerging variants. We observed a more complex tree topology for influenza B, where new lineages emerged from deep branches and evolved toward multiple directions (Figure 2, panel B). In addition, multiple influenza B variants co-circulated for a median of 6 years (range 2–15 years) before being displaced by new variants.

Of the 983 circulating strains examined, 8 (maximum 2 per season) influenza A(H3N2) and 12 (maximum 4 per season) influenza B viruses were regarded as far away from any vaccine strains recommended during the 17-year study period. The pairwise HA amino acid distances of these strains were more than twice the SD from the mean of the related clusters (Technical Appendix Figures 1, 2), and they were distant from all vaccine strains based on the phylogenetic tree topology.

### Matching Between Circulating and Vaccine Strains

#### Influenza A(H3N2)

**Figure 3:**
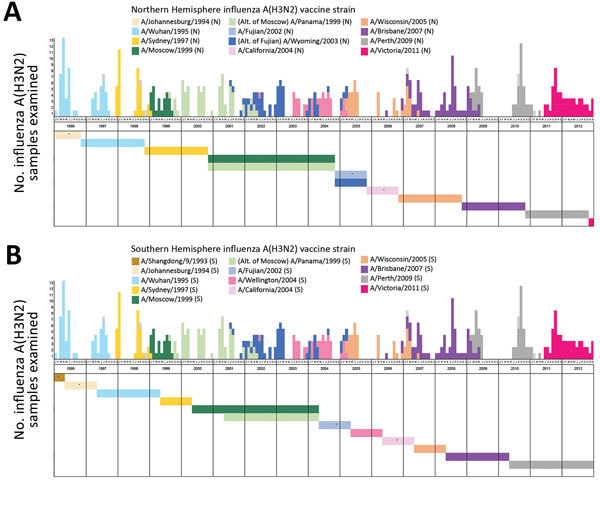
Matching between circulating and vaccine strains of influenza A(H3N2), Hong Kong, China, 1996–2012. Each circulating virus was assigned on the basis of full-length hemagglutinin amino acid distance and phylogenetic tree topology to the closest World Health Organization–recommended influenza A(H3N2) vaccine strain for Northern Hemisphere (A) and Southern Hemisphere (B) vaccines. Closely matched viruses are labeled with the same color. The circulating strains with no closest vaccine strain identified as defined in the Methods are indicated by dotted boxes. Horizontal color bars indicate the protection period of the corresponding vaccine strain. Asterisks indicate vaccine strains without closely matched circulating viruses. The protection period of Northern Hemisphere vaccines was defined as from November through the following October and of Southern Hemisphere vaccines from May through the following April. All the alternative vaccine strains were analyzed, and those with different results are shown.

We assessed the temporal relationship between circulating H3N2 viruses collected in this study and their closest Northern Hemisphere vaccine strain based on HA amino acid sequence comparisons (Figure 3, panel A). Most Northern hemisphere vaccine-like strains (9/11) had circulated in Hong Kong for >1 year before WHO first recommended them for inclusion in the Northern Hemisphere vaccine. We found co-circulation of multiple strains in 17 (50.0%) of the 34 seasons. In 13 (38.2%) seasons, at least a portion of the circulating viruses were closely matched with the contemporary vaccine strain. However, in only 7 (20.6%) seasons, the closely matched portion accounted for the >50% of circulating viruses examined. A full match was achieved in only 2 (5.9%) seasons. We determined the proportion of circulating viruses that were genetically closely matched with vaccine strains based on HA amino acid sequence comparison (Table), which should not be taken as vaccine effectiveness because cross-protection between mismatched strains can occur.

**Table:** Proportion of circulating influenza viruses with HA amino acid sequences closely matched with vaccine strains, Hong Kong, China, 1996–2012*

Year, season	Influenza A(H3N2) vaccine strains, %†			Influenza B vaccine strains, %†	
	Northern Hemisphere	Southern Hemisphere		Northern Hemisphere	Southern Hemisphere
1996					
Winter	None‡	None‡		All§	All§
Summer	None‡	None‡		77.8	77.8
1997					
Winter	All§	None‡		14.7	None‡
Summer	94.4	94.4		None‡	None‡
1998					
Winter	None‡	None‡		None‡	None‡
Summer	None‡	None‡		None‡	None‡
1999					
Winter	18.8	None‡		6.3	6.3
Summer	None‡	None‡		None‡	None‡
2000					
Winter	None‡	None‡		None‡	None‡
Summer	None‡	None‡		None‡	None‡
2001					
Winter	All§¶	None‡		None‡	None‡
Summer	84.6¶	84.6¶		None‡	25.0
2002					
Winter	41.2¶	41.2¶		None‡	None‡
Summer	6.7	6.7		12.5	12.5
2003					
Winter	None‡	None‡		73.3	6.7
Summer	None‡	None‡		None‡	None‡
2004					
Winter	None‡	None‡		None‡	None‡
Summer	None‡	None‡		None‡	None‡
2005					
Winter	31.3#	None‡		29.4	None‡
Summer	None‡	None‡		12.5	12.5
2006					
Winter	None‡	None‡		None‡	None‡
Summer	None‡	None‡		None‡	90.0
2007					
Winter	52.4	None‡		9.1	9.1
Summer	6.7	6.7		None‡	None‡
2008					
Winter	None‡	None‡		22.2	22.2
Summer	None‡	All§		None‡	None‡
2009					
Winter	52.2	52.2		None‡	None‡
Summer**	22.2	22.2		None‡	None‡
2010					
Winter	None‡	None‡		66.7	None‡
Summer	None‡	92.9		26.7	26.7
2011					
Winter	87.5	87.5		64.3	64.3
Summer	None‡	None‡		37.5	37.5
2012					
Winter	None‡	None‡		31.0	31.0
Summer	None‡	None‡		None‡	None‡

*Calendar months belonging to winter or summer influenza seasons were based on the average number of cases admitted per month during the study period (Figure 1). Winter seasons were November of previous year through April of current year; influenza usually peaks in February and March. Summer seasons were May–October; influenza usually peaks in July and August. HA, hemagglutinin. †Co-circulation of multiple strains with a certain percentage closely matched the contemporary vaccine strain based on HA amino acid sequence comparison. ‡None of the circulating viruses examined were closely matched with the contemporary vaccine strain based on HA amino acid sequence comparison. §All circulating viruses examined were closely matched with the contemporary vaccine strain based on HA amino acid sequence comparison. ¶A/Panama/1999, an alternate to A/Moscow/99, closely matched the circulating strains of 2001 winter and summer seasons and 2002 winter season. #The World Health Organization recommended 2 A(H3N2) vaccine strains for the Northern Hemisphere in 2005, one of which, A/Wyoming/2003, closely matched a portion of the viruses circulating during the 2005 winter season. **Dominated by influenza A(H1N1)pdm09.

We repeated the same analysis with respect to Southern Hemisphere vaccine strains. Similarly, most (8/11) Southern Hemisphere H3N2 virus vaccine strains had circulated in Hong Kong for >1 year before WHO first recommended them for inclusion in the Southern Hemisphere vaccine. A similar picture on matching was revealed (Figure 3, panel B). In 10 (29.4%) of the 34 seasons, at least a portion of the circulating viruses were closely matched with the contemporary vaccine strain. However, only in 5 (14.7%) seasons did the matched portion account for the >50% of circulating viruses examined.

#### Influenza B

**Figure 4:**
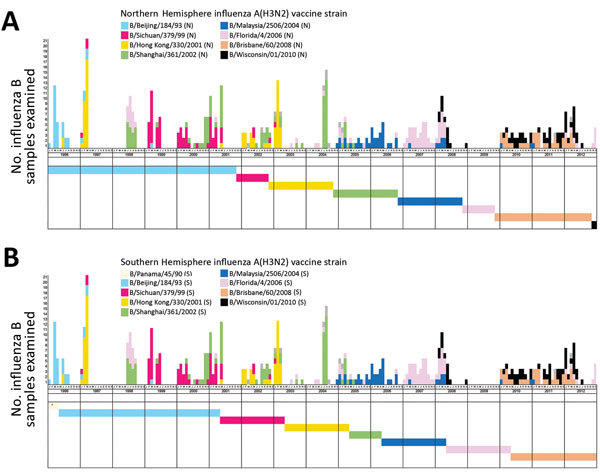
Matching between circulating and vaccine strains of influenza B, Hong Kong, China, 1996–2012. Each circulating virus was assigned on the basis of full-length hemagglutinin amino acid distances and phylogenetic tree topology to the closest World Health Organization–recommended influenza B vaccine strain for Northern Hemisphere (A) and Southern Hemisphere (B) vaccines. Closely matched viruses are labeled with the same color. The circulating strains with no closest vaccine strain identified as defined in the Methods are indicated by dotted boxes. Horizontal color bars indicate the protection period of the corresponding vaccine strain. Asterisks indicate vaccine strains without closely matched circulating viruses. The protection period of Northern Hemisphere (A) vaccines was defined as from November through the following October and of Southern Hemisphere (B) vaccines from May through the following April. All the alternative vaccine strains were analyzed, and those with different results are shown.

We assessed the temporal relationship between circulating influenza B viruses and their closely matched Northern Hemisphere vaccine strains based on HA amino acid sequence comparisons (Figure 4, panel A). Most (6/7) of the Northern Hemisphere influenza B vaccine–like circulating strains had been found in Hong Kong >1 year before WHO first recommended them as vaccine strains. Co-circulation of multiple strains was found in 26 (76.5%) of the 34 seasons examined. In 15 (44.1%) seasons, at least some of the circulating viruses were closely matched with the contemporary Northern Hemisphere vaccine strain. However, in only 5 (14.7%) seasons did the matched portion account for the >50% of circulating viruses examined.

Analysis of Southern Hemisphere vaccine strains showed a similar result (Figure 4, panel B). Most (5/6) of the vaccine-like strains were found in Hong Kong >1 year before WHO first recommended them as vaccine strains. Again, we found matched strains in 14 (41.2%) seasons, but in only 4 (11.8%) seasons did the matched viruses account for the >50% of viruses examined.

### Strain Drifting between Winter and Summer Seasons

#### Influenza A(H3N2)

In 7 of the 17 years (2002, 2003, 2004, 2005, 2007, 2009, and 2011), the predominant H3N2 virus strain in summer differed from that in the preceding winter. We further examined whether administration of an additional Southern Hemisphere vaccine at the beginning of summer (around May) could achieve better vaccine matching. In 5 of the 7 years, content of newly available Southern Hemisphere vaccines was identical to the preceding Northern Hemisphere vaccine composition. In 2004 and 2005, although another strain was chosen for the Southern Hemisphere vaccine, the new vaccine strain was not closely matched with circulating viruses at that time. These results suggested that additional administration of Southern Hemisphere vaccines at the beginning of summer would not have improved the immunity against the circulating H3N2 strains.

#### Influenza B

In 6 of the 17 years (2000, 2002, 2004, 2008, 2010, and 2011), a drift occurred in the predominant influenza B variant from winter to summer seasons. Similarly, we examined the value of an additional Southern Hemisphere vaccine administered at the beginning of summer and again found no benefit. In 5 of the 6 drift years, the newly recommended Southern Hemisphere vaccine composition was identical to the previous Northern Hemisphere vaccine. In 2008, although the Southern Hemisphere vaccine contained a new virus (Yamagata, B/Florida/4/2006) that closely matched the circulating viruses detected in the first half (winter season) of the same year, the circulating strain was soon replaced by another influenza B variant not covered in the recommended Southern Hemisphere vaccine.

## Discussion

This study focused on the vaccine matching of influenza A(H3N2) and influenza B viruses because antigenic drift frequently occurs in these viruses, leading to changes in vaccine recommendation, more so than for influenza A(H1N1) virus (*7*). Antigenic characterization of influenza viruses, and thus the assessment of vaccine matching, has traditionally been based on hemagglutination inhibition (HI) assay. HI measures the ability of specific antibodies, typically reference ferret antiserum, to inhibit the binding of virus HA to receptors on erythrocytes. However, H3N2 virus variants from the past 3 decades have progressively displayed reduced avidity to erythrocytes of commonly used animal species, making the results of HI assays difficult to interpret (*8*,*18*). In this study, we analyzed the amino acid sequence of HA to infer vaccine matching because genetic and antigenic characteristics of influenza viruses display remarkable correspondence, and both carry a strong correlation with vaccine effectiveness (*19*–*21*). The molecular phylogenetic approach used enables direct analyses of viruses in clinical samples rather than relying on cell culture–propagated isolates. Furthermore, the molecular approach does not require influenza variant–specific antiserum and is more feasible for large-scale epidemiologic surveys. One caution in interpreting molecular results is that influenza vaccines might still display cross-protection efficacy against circulating variants that are regarded as not closely matched to the vaccine strains based on genetic comparison. Nevertheless, our approach to define matching between vaccine and circulating strains would be sufficient to guide the selection of Northern and Southern Hemisphere vaccines and to inform the potential benefit of a biannual vaccination strategy using both Northern and Southern Hemisphere vaccines on the background of influenza epidemiology faced by subtropical countries.

Given the extensive amount of travel between Hong Kong and neighboring East Asia cities, we expect a substantial sharing of viruses detected in Hong Kong with the region. Although the proportion of infectious travelers is difficult to estimate, a study from Xiamen International Airport (Xiamen, China) in 2015–2016 found that ≈8/10,000 incoming travelers had influenza-like illness, of whom 21% tested positive for influenza (*22*). The fact that Hong Kong shares a similar, though not always synchronized, pool of influenza viruses with the neighboring East and Southeast Asian cities is supported by a study from Bahl et al. (*23*) and sequence information from Nextstrain (https://nextstrain.org). East and Southeast Asia are the sources of new genetic variants of H3N2 virus, which seed epidemics worldwide (*24*–*28*). In line with this, we demonstrated that most of the H3N2 virus strains selected for inclusion in influenza vaccines could be detected in East Asia >1 year before recommendation by WHO. Most of these newly emerged strains predominated for 2 years, covering 4 influenza seasons in this region. Although vaccine compositions were reviewed twice a year, when vaccines incorporating newly emerged strains were made available to the market, often another newly drifted influenza strain had become predominant in the region. Based on our analysis covering 17 years with 34 influenza seasons, the probability of a season having the contemporary vaccine closely matched with >50% of the circulating viruses was only 20.6%, and if we aimed at matching >70% of circulating viruses, the probability was further reduced to 14.7%. As reflected in a recent study on vaccine effectiveness against hospitalization among children in Hong Kong, efficacy against H3N2 virus was on average 36.6% (*29*). 

Because Hong Kong, like many other subtropical cities, faces multiple influenza peaks annually, we raised the question whether Southern Hemisphere influenza vaccines would provide timely and better vaccine matching. However, we observed that most of the time composition of the Southern Hemisphere vaccine is the same as the preceding Northern Hemisphere vaccine and thus made no difference in vaccine matching. Furthermore, according to the current production schedule, Southern Hemisphere vaccines are made available in May, just after the period with highest influenza activity in Hong Kong (January–March). Therefore, to administer influenza vaccine in May requires vaccines with efficacy lasting for almost 12 months, which is not a demonstrated property of current vaccines.

Given the comparable duration of winter and summer influenza seasons in Hong Kong, the next question was whether biannual vaccination with Northern and Southern Hemisphere vaccines administered before the winter and summer seasons, respectively, would improve immunity against circulating strains. In early 2015, Hong Kong faced a strong winter influenza peak associated with a newly drifted variant, influenza A(H3N2)/Switzerland, which was not incorporated into the Northern Hemisphere vaccine. In view of a possible strong summer peak, an ad hoc vaccination campaign was conducted in May using the newly available Southern Hemisphere vaccine that incorporated the new variant. However, willingness to accept the vaccine was low among healthcare professionals, and a major proportion expressed concern (*30*). Furthermore, in recent years, concern has surfaced that repeated influenza vaccination might negatively affect current vaccine effectiveness (*31*–*33*). Although a recent meta-analysis does not support such a claim (*34*), this possibility should not be ignored when considering biannual vaccination. Although the cost-effectiveness and feasibility of a biannual vaccination strategy is yet to be assessed, our analysis does not support that an additional vaccine before summer seasons can improve matching. We observed a substantial chance of drift in predominant influenza viruses from season to season (41.2% for H3N2 and 35.3% for influenza B), and the newly available Southern Hemisphere vaccines typically had the same strain composition as the preceding Northern Hemisphere vaccine or the updated Southern Hemisphere composition still did not achieve a close match with the then-circulating viruses.

East Asia and other subtropical regions face different challenges in achieving close vaccine matching for influenza A(H3N2) and influenza B. For H3N2 virus, the forthcoming predominant strain can be identified from a subtropical region–based surveillance network. The newly emerged strains usually predominate for 1–2 years, which cover 4 influenza seasons in this region. If recommendation on vaccine composition followed by manufacturing is accomplished within a few months, a close match can be achieved in the subsequent 2–3 seasons to provide the best possible vaccine effectiveness. This streamlined workflow should be practicable with current logistics and production provided that a sustainable market can be established in the region.

The challenge posed by influenza B is completely different. Although Bedford et al. suggested that East and Southeast Asia play limited roles in disseminating new variants of influenza B (*24*), we observed that most of the influenza B strains could be found in East Asia long before they are selected as vaccine strains. East Asia is therefore also an important site for identifying emergent influenza B strains. However, co-circulation and multiple reintroductions of existing and emerging influenza strains complicate selection of vaccine strains. Incorporating both lineages of influenza B into the quadrivalent vaccine might be able to solve at least part of the problem. In either case, a next-generation influenza vaccine should be designed to provide broad cross-protection against different antigenic variants (*35*).

East Asia is an important contributor to the influenza virus surveillance program. However, vaccine strains incorporated in the Northern and Southern Hemisphere influenza vaccines frequently do not match the contemporary circulating viruses in this region. Even biannual vaccination is unlikely to improve vaccine matching. A specific strategy is urgently needed to select and produce influenza vaccines targeting the tropical and subtropical regions.

Technical Appendix GenBank accession numbers of hemagglutinin sequences of circulating influenza A and B strains, Hong Kong, China, 1996–2012; pairwise hemagglutinin amino acid distances between circulating and vaccine strains of influenza A(H3N2) and influenza B.
