# Supplementary material for: Frequent Genetic Mismatch between Vaccine Strains and Circulating Seasonal Influenza Viruses, Hong Kong, China, 1996–2012
Source: Emerg Infect Dis. 2018 Oct;24(10):1825–34. doi: 10.3201/eid2410.180652 (PMC6154132; doi:10.3201/eid2410.180652)
Supplement: Technical Appendix — GenBank accession numbers of hemagglutinin sequences of circulating influenza A and B strains, Hong Kong, China, 1996–2012; pairwise hemagglutinin amino acid distances between circulating and vaccine strains of influenza A(H3N2) and influenza B. [file 18-0652-Techapp-s1.pdf]

Article DOI: https://doi.org/10.3201/eid2410.180652

Frequent Genetic Mismatch between Vaccine Strains and Circulating Seasonal Influenza Viruses, Hong Kong, China,1996–2012

Technical Appendix

Technical Appendix Table. GenBank accession numbers of hemagglutinin sequences of circulating influenza A and B strains, Hong Kong, China, 1996–2012*

GenBank accession no.	Type	Isolate ID	Collection date
EU516331	FluA_H3N2_HA	A/Hong Kong/CUHK22910/2004(H3N2)	Mar-2004
EU856814	FluA_H3N2_HA	A/Hong Kong/CUHK10026/2005(H3N2)	Apr-2005
EU856815	FluA_H3N2_HA	A/Hong Kong/CUHK10100/1999(H3N2)	Jan-1999
EU856816	FluA_H3N2_HA	A/Hong Kong/CUHK10132/1999(H3N2)	Jan-1999
EU856817	FluA_H3N2_HA	A/Hong Kong/CUHK10133/1999(H3N2)	Jan-1999
EU856818	FluA_H3N2_HA	A/Hong Kong/CUHK10151/1999(H3N2)	Jan-1999
EU856819	FluA_H3N2_HA	A/Hong Kong/CUHK10186/1998(H3N2)	Jan-1998
EU856820	FluA_H3N2_HA	A/Hong Kong/CUHK10216/1998(H3N2)	Jan-1998
EU856821	FluA_H3N2_HA	A/Hong Kong/CUHK10276/1998(H3N2)	Jan-1998
EU856822	FluA_H3N2_HA	A/Hong Kong/CUHK10297/2005(H3N2)	Apr-2005
EU856824	FluA_H3N2_HA	A/Hong Kong/CUHK10554/1998(H3N2)	Jan-1998
EU856825	FluA_H3N2_HA	A/Hong Kong/CUHK10591/2005(H3N2)	Apr-2005
EU856826	FluA_H3N2_HA	A/Hong Kong/CUHK10632/1998(H3N2)	Jan-1998
EU856827	FluA_H3N2_HA	A/Hong Kong/CUHK10660/1998(H3N2)	Jan-1998
EU856829	FluA_H3N2_HA	A/Hong Kong/CUHK010954/1998(H3N2)	Jan-1998
EU856830	FluA_H3N2_HA	A/Hong Kong/CUHK10958/1998(H3N2)	Jan-1998
EU856833	FluA_H3N2_HA	A/Hong Kong/CUHK11230/1999(H3N2)	Feb-1999
EU856834	FluA_H3N2_HA	A/Hong Kong/CUHK11237/1999(H3N2)	Feb-1999
EU856836	FluA_H3N2_HA	A/Hong Kong/CUHK11255/1999(H3N2)	Feb-1999
EU856837	FluA_H3N2_HA	A/Hong Kong/CUHK11949/2005(H3N2)	Apr-2005
EU856839	FluA_H3N2_HA	A/Hong Kong/CUHK12160/1997(H3N2)	May-1997
EU856840	FluA_H3N2_HA	A/Hong Kong/CUHK12312/1999(H3N2)	Mar-1999
EU856841	FluA_H3N2_HA	A/Hong Kong/CUHK12316/2005(H3N2)	May-2005
EU856842	FluA_H3N2_HA	A/Hong Kong/CUHK12320/2005(H3N2)	May-2005
EU856843	FluA_H3N2_HA	A/Hong Kong/CUHK12420/2005(H3N2)	May-2005
EU856844	FluA_H3N2_HA	A/Hong Kong/CUHK12425/2005(H3N2)	May-2005
EU856845	FluA_H3N2_HA	A/Hong Kong/CUHK12563/1997(H3N2)	Jun-1997
EU856846	FluA_H3N2_HA	A/Hong Kong/CUHK12566/1997(H3N2)	Jun-1997
EU856847	FluA_H3N2_HA	A/Hong Kong/CUHK12572/1999(H3N2)	Apr-1999
EU856848	FluA_H3N2_HA	A/Hong Kong/CUHK12580/1999(H3N2)	Apr-1999
EU856849	FluA_H3N2_HA	A/Hong Kong/CUHK12626/1997(H3N2)	Jun-1997
EU856850	FluA_H3N2_HA	A/Hong Kong/CUHK12794/1997(H3N2)	Jun-1997
EU856851	FluA_H3N2_HA	A/Hong Kong/CUHK12897/1999(H3N2)	Apr-1999
EU856852	FluA_H3N2_HA	A/Hong Kong/CUHK13033/2001(H3N2)	Mar-2001
EU856853	FluA_H3N2_HA	A/Hong Kong/CUHK13048/2001(H3N2)	Mar-2001
EU856854	FluA_H3N2_HA	A/Hong Kong/CUHK13126/2004(H3N2)	Feb-2004
EU856855	FluA_H3N2_HA	A/Hong Kong/CUHK13172/2001(H3N2)	Mar-2001
EU856856	FluA_H3N2_HA	A/Hong Kong/CUHK13216/2002(H3N2)	Mar-2002
EU856857	FluA_H3N2_HA	A/Hong Kong/CUHK13249/2002(H3N2)	Mar-2002
EU856858	FluA_H3N2_HA	A/Hong Kong/CUHK13278/2002(H3N2)	Mar-2002
EU856859	FluA_H3N2_HA	A/Hong Kong/CUHK13299/2003(H3N2)	Mar-2003
EU856860	FluA_H3N2_HA	A/Hong Kong/CUHK13339/2003(H3N2)	Mar-2003
EU856861	FluA_H3N2_HA	A/Hong Kong/CUHK13434/2003(H3N2)	Mar-2003
EU856862	FluA_H3N2_HA	A/Hong Kong/CUHK13483/2003(H3N2)	Mar-2003
EU856863	FluA_H3N2_HA	A/Hong Kong/CUHK13510/2001(H3N2)	Mar-2001
EU856864	FluA_H3N2_HA	A/Hong Kong/CUHK13527/2003(H3N2)	Mar-2003
EU856865	FluA_H3N2_HA	A/Hong Kong/CUHK13658/2001(H3N2)	Mar-2001

GenBank accession no.	Type	Isolate ID	Collection date
EU856866	FluA_H3N2_HA	A/Hong Kong/CUHK13763/1999(H3N2)	May-1999
EU856867	FluA_H3N2_HA	A/Hong Kong/CUHK14627/1999(H3N2)	Jun-1999
EU856868	FluA_H3N2_HA	A/Hong Kong/CUHK14672/1999(H3N2)	Jun-1999
EU856869	FluA_H3N2_HA	A/Hong Kong/CUHK14875/1999(H3N2)	Jun-1999
EU856870	FluA_H3N2_HA	A/Hong Kong/CUHK14999/2005(H3N2)	Jun-2005
EU856871	FluA_H3N2_HA	A/Hong Kong/CUHK16614/1998(H3N2)	Jul-1998
EU856872	FluA_H3N2_HA	A/Hong Kong/CUHK17464/1998(H3N2)	Aug-1998
EU856873	FluA_H3N2_HA	A/Hong Kong/CUHK17603/1998(H3N2)	Aug-1998
EU856875	FluA_H3N2_HA	A/Hong Kong/CUHK17697/1998(H3N2)	Aug-1998
EU856876	FluA_H3N2_HA	A/Hong Kong/CUHK17704/1998(H3N2)	Aug-1998
EU856877	FluA_H3N2_HA	A/Hong Kong/CUHK17707/1998(H3N2)	Aug-1998
EU856878	FluA_H3N2_HA	A/Hong Kong/CUHK17872/1998(H3N2)	Aug-1998
EU856879	FluA_H3N2_HA	A/Hong Kong/CUHK18036/1998(H3N2)	Aug-1998
EU856880	FluA_H3N2_HA	A/Hong Kong/CUHK18194/1998(H3N2)	Sep-1998
EU856881	FluA_H3N2_HA	A/Hong Kong/CUHK18218/1998(H3N2)	Sep-1998
EU856882	FluA_H3N2_HA	A/Hong Kong/CUHK18230/1998(H3N2)	Sep-1998
EU856883	FluA_H3N2_HA	A/Hong Kong/CUHK18351/1998(H3N2)	Sep-1998
EU856884	FluA_H3N2_HA	A/Hong Kong/CUHK18358/1998(H3N2)	Sep-1998
EU856885	FluA_H3N2_HA	A/Hong Kong/CUHK18610/1998(H3N2)	Sep-1998
EU856886	FluA_H3N2_HA	A/Hong Kong/CUHK18886/1998(H3N2)	Oct-1998
EU856887	FluA_H3N2_HA	A/Hong Kong/CUHK19579/1998(H3N2)	Nov-1998
EU856888	FluA_H3N2_HA	A/Hong Kong/CUHK20010/1997(H3N2)	Jun-1997
EU856889	FluA_H3N2_HA	A/Hong Kong/CUHK20169/2000(H3N2)	Jan-2000
EU856890	FluA_H3N2_HA	A/Hong Kong/CUHK20173/2000(H3N2)	Jan-2000
EU856891	FluA_H3N2_HA	A/Hong Kong/CUHK20199/1997(H3N2)	Jul-1997
EU856892	FluA_H3N2_HA	A/Hong Kong/CUHK20200/2000(H3N2)	Jan-2000
EU856893	FluA_H3N2_HA	A/Hong Kong/CUHK20213/1997(H3N2)	Jul-1997
EU856894	FluA_H3N2_HA	A/Hong Kong/CUHK20217/1997(H3N2)	Jul-1997
EU856895	FluA_H3N2_HA	A/Hong Kong/CUHK20236/1997(H3N2)	Jul-1997
EU856896	FluA_H3N2_HA	A/Hong Kong/CUHK20292/2000(H3N2)	Jan-2000
EU856897	FluA_H3N2_HA	A/Hong Kong/CUHK20294/2000(H3N2)	Jan-2000
EU856898	FluA_H3N2_HA	A/Hong Kong/CUHK20300/2000(H3N2)	Jan-2000
EU856899	FluA_H3N2_HA	A/Hong Kong/CUHK20320/1997(H3N2)	Jul-1997
EU856900	FluA_H3N2_HA	A/Hong Kong/CUHK20523/1997(H3N2)	Jul-1997
EU856901	FluA_H3N2_HA	A/Hong Kong/CUHK20731/1997(H3N2)	Jul-1997
EU856902	FluA_H3N2_HA	A/Hong Kong/CUHK20992/1997(H3N2)	Aug-1997
EU856903	FluA_H3N2_HA	A/Hong Kong/CUHK21124/2000(H3N2)	Feb-2000
EU856905	FluA_H3N2_HA	A/Hong Kong/CUHK21132/2000(H3N2)	Feb-2000
EU856906	FluA_H3N2_HA	A/Hong Kong/CUHK21164/2004(H3N2)	Feb-2004
EU856907	FluA_H3N2_HA	A/Hong Kong/CUHK21226/2001(H3N2)	Apr-2001
EU856908	FluA_H3N2_HA	A/Hong Kong/CUHK21250/2000(H3N2)	Feb-2000
EU856909	FluA_H3N2_HA	A/Hong Kong/CUHK21259/2004(H3N2)	Feb-2004
EU856910	FluA_H3N2_HA	A/Hong Kong/CUHK21421/2001(H3N2)	Apr-2001
EU856911	FluA_H3N2_HA	A/Hong Kong/CUHK21582/2002(H3N2)	Apr-2002
EU856912	FluA_H3N2_HA	A/Hong Kong/CUHK21675/2001(H3N2)	Apr-2001
EU856913	FluA_H3N2_HA	A/Hong Kong/CUHK21713/2002(H3N2)	Apr-2002
EU856914	FluA_H3N2_HA	A/Hong Kong/CUHK21733/2001(H3N2)	Apr-2001
EU856915	FluA_H3N2_HA	A/Hong Kong/CUHK21734/2001(H3N2)	Apr-2001
EU856916	FluA_H3N2_HA	A/Hong Kong/CUHK21742/2002(H3N2)	Apr-2002
EU856918	FluA_H3N2_HA	A/Hong Kong/CUHK021932/2001(H3N2)	Apr-2001
EU856919	FluA_H3N2_HA	A/Hong Kong/CUHK21957/2002(H3N2)	Apr-2002
EU856920	FluA_H3N2_HA	A/Hong Kong/CUHK22013/1997(H3N2)	Aug-1997
EU856921	FluA_H3N2_HA	A/Hong Kong/CUHK22048/1997(H3N2)	Aug-1997
EU856922	FluA_H3N2_HA	A/Hong Kong/CUHK22072/2000(H3N2)	Mar-2000
EU856923	FluA_H3N2_HA	A/Hong Kong/CUHK22078/2000(H3N2)	Mar-2000
EU856924	FluA_H3N2_HA	A/Hong Kong/CUHK22087/2000(H3N2)	Mar-2000
EU856925	FluA_H3N2_HA	A/Hong Kong/CUHK22162/2000(H3N2)	Mar-2000
EU856926	FluA_H3N2_HA	A/Hong Kong/CUHK22163/2000(H3N2)	Mar-2000
EU856928	FluA_H3N2_HA	A/Hong Kong/CUHK22510/2004(H3N2)	Mar-2004
EU856929	FluA_H3N2_HA	A/Hong Kong/CUHK22567/2004(H3N2)	Mar-2004
EU856930	FluA_H3N2_HA	A/Hong Kong/CUHK22644/2001(H3N2)	May-2001
EU856931	FluA_H3N2_HA	A/Hong Kong/CUHK22736/1997(H3N2)	Sep-1997
EU856932	FluA_H3N2_HA	A/Hong Kong/CUHK22888/2001(H3N2)	May-2001
EU856933	FluA_H3N2_HA	A/Hong Kong/CUHK23111/2002(H3N2)	May-2002
EU856934	FluA_H3N2_HA	A/Hong Kong/CUHK23113/2002(H3N2)	May-2002
EU856935	FluA_H3N2_HA	A/Hong Kong/CUHK23162/2002(H3N2)	May-2002
EU856936	FluA_H3N2_HA	A/Hong Kong/CUHK23180/2002(H3N2)	May-2002
EU856937	FluA_H3N2_HA	A/Hong Kong/CUHK24044/2002(H3N2)	Jun-2002
EU856938	FluA_H3N2_HA	A/Hong Kong/CUHK24054/2002(H3N2)	Jun-2002

GenBank accession no.	Type	Isolate ID	Collection date
EU856939	FluA_H3N2_HA	A/Hong Kong/CUHK24114/2002(H3N2)	Jun-2002
EU856940	FluA_H3N2_HA	A/Hong Kong/CUHK24167/2002(H3N2)	Jun-2002
EU856942	FluA_H3N2_HA	A/Hong Kong/CUHK24289/2004(H3N2)	Apr-2004
EU856944	FluA_H3N2_HA	A/Hong Kong/CUHK24447/2004(H3N2)	Apr-2004
EU856945	FluA_H3N2_HA	A/Hong Kong/CUHK24510/2001(H3N2)	Jul-2001
EU856946	FluA_H3N2_HA	A/Hong Kong/CUHK24749/2001(H3N2)	Jul-2001
EU856947	FluA_H3N2_HA	A/Hong Kong/CUHK24760/2001(H3N2)	Jul-2001
EU856948	FluA_H3N2_HA	A/Hong Kong/CUHK24825/2001(H3N2)	Jul-2001
EU856949	FluA_H3N2_HA	A/Hong Kong/CUHK24882/2001(H3N2)	Jul-2001
EU856950	FluA_H3N2_HA	A/Hong Kong/CUHK24985/2001(H3N2)	Jul-2001
EU856951	FluA_H3N2_HA	A/Hong Kong/CUHK26805/2000(H3N2)	Jul-2000
EU856952	FluA_H3N2_HA	A/Hong Kong/CUHK26846/2000(H3N2)	Jul-2000
EU856953	FluA_H3N2_HA	A/Hong Kong/CUHK26907/2000(H3N2)	Jul-2000
EU856954	FluA_H3N2_HA	A/Hong Kong/CUHK26969/2000(H3N2)	Jul-2000
EU856956	FluA_H3N2_HA	A/Hong Kong/CUHK27157/2000(H3N2)	Aug-2000
EU856957	FluA_H3N2_HA	A/Hong Kong/CUHK27183/2000(H3N2)	Aug-2000
EU856959	FluA_H3N2_HA	A/Hong Kong/CUHK27374/2000(H3N2)	Aug-2000
EU856960	FluA_H3N2_HA	A/Hong Kong/CUHK28038/2000(H3N2)	Sep-2000
EU856961	FluA_H3N2_HA	A/Hong Kong/CUHK28040/2000(H3N2)	Sep-2000
EU856965	FluA_H3N2_HA	A/Hong Kong/CUHK30314/1999(H3N2)	Jul-1999
EU856966	FluA_H3N2_HA	A/Hong Kong/CUHK30419/1999(H3N2)	Jul-1999
EU856968	FluA_H3N2_HA	A/Hong Kong/CUHK30605/1999(H3N2)	Jul-1999
EU856969	FluA_H3N2_HA	A/Hong Kong/CUHK31421/1999(H3N2)	Aug-1999
EU856970	FluA_H3N2_HA	A/Hong Kong/CUHK31448/1999(H3N2)	Aug-1999
EU856971	FluA_H3N2_HA	A/Hong Kong/CUHK31490/1999(H3N2)	Aug-1999
EU856972	FluA_H3N2_HA	A/Hong Kong/CUHK31510/1999(H3N2)	Aug-1999
EU856973	FluA_H3N2_HA	A/Hong Kong/CUHK32796/1999(H3N2)	Sep-1999
EU856974	FluA_H3N2_HA	A/Hong Kong/CUHK33047/2002(H3N2)	Jul-2002
EU856975	FluA_H3N2_HA	A/Hong Kong/CUHK33079/2002(H3N2)	Jul-2002
EU856976	FluA_H3N2_HA	A/Hong Kong/CUHK33106/2002(H3N2)	Jul-2002
EU856977	FluA_H3N2_HA	A/Hong Kong/CUHK33199/2002(H3N2)	Jul-2002
EU856978	FluA_H3N2_HA	A/Hong Kong/CUHK33316/2001(H3N2)	Aug-2001
EU856979	FluA_H3N2_HA	A/Hong Kong/CUHK33406/2001(H3N2)	Aug-2001
EU856980	FluA_H3N2_HA	A/Hong Kong/CUHK33416/2001(H3N2)	Aug-2001
EU856981	FluA_H3N2_HA	A/Hong Kong/CUHK33418/2001(H3N2)	Aug-2001
EU856982	FluA_H3N2_HA	A/Hong Kong/CUHK33677/2004(H3N2)	May-2004
EU856983	FluA_H3N2_HA	A/Hong Kong/CUHK33829/1999(H3N2)	Oct-1999
EU856984	FluA_H3N2_HA	A/Hong Kong/CUHK33851/2004(H3N2)	May-2004
EU856986	FluA_H3N2_HA	A/Hong Kong/CUHK33894/2004(H3N2)	May-2004
EU856987	FluA_H3N2_HA	A/Hong Kong/CUHK33915/1999(H3N2)	Oct-1999
EU856988	FluA_H3N2_HA	A/Hong Kong/CUHK34114/2001(H3N2)	Aug-2001
EU856989	FluA_H3N2_HA	A/Hong Kong/CUHK34175/2002(H3N2)	Aug-2002
EU856990	FluA_H3N2_HA	A/Hong Kong/CUHK34193/2002(H3N2)	Feb-2002
EU856992	FluA_H3N2_HA	A/Hong Kong/CUHK35011/2004(H3N2)	Jun-2004
EU856993	FluA_H3N2_HA	A/Hong Kong/CUHK35013/2004(H3N2)	Jun-2004
EU856994	FluA_H3N2_HA	A/Hong Kong/CUHK35117/2004(H3N2)	Jun-2004
EU856995	FluA_H3N2_HA	A/Hong Kong/CUHK35435/2004(H3N2)	Jun-2004
EU856996	FluA_H3N2_HA	A/Hong Kong/CUHK40022/2005(H3N2)	Jun-2005
EU856997	FluA_H3N2_HA	A/Hong Kong/CUHK40139/2005(H3N2)	Jun-2005
EU857001	FluA_H3N2_HA	A/Hong Kong/CUHK41114/1997(H3N2)	Dec-1997
EU857002	FluA_H3N2_HA	A/Hong Kong/CUHK41222/1997(H3N2)	Dec-1997
EU857003	FluA_H3N2_HA	A/Hong Kong/CUHK41459/1997(H3N2)	Dec-1997
EU857004	FluA_H3N2_HA	A/Hong Kong/CUHK4147/1997(H3N2)	Mar-1997
EU857006	FluA_H3N2_HA	A/Hong Kong/CUHK41757/1997(H3N2)	Dec-1997
EU857007	FluA_H3N2_HA	A/Hong Kong/CUHK42241/2005(H3N2)	Jul-2005
EU857008	FluA_H3N2_HA	A/Hong Kong/CUHK4245/1997(H3N2)	Mar-1997
EU857009	FluA_H3N2_HA	A/Hong Kong/CUHK42644/2005(H3N2)	Jul-2005
EU857010	FluA_H3N2_HA	A/Hong Kong/CUHK42763/2005(H3N2)	Jul-2005
EU857011	FluA_H3N2_HA	A/Hong Kong/CUHK43751/2005(H3N2)	Jul-2005
EU857012	FluA_H3N2_HA	A/Hong Kong/CUHK4391/1997(H3N2)	Mar-1997
EU857013	FluA_H3N2_HA	A/Hong Kong/CUHK45130/2006(H3N2)	Dec-2006
EU857014	FluA_H3N2_HA	A/Hong Kong/CUHK4529/1997(H3N2)	Apr-1997
EU857018	FluA_H3N2_HA	A/Hong Kong/CUHK50080/2001(H3N2)	Nov-2001
EU857019	FluA_H3N2_HA	A/Hong Kong/CUHK50200/2002(H3N2)	Oct-2002
EU857021	FluA_H3N2_HA	A/Hong Kong/CUHK50372/2004(H3N2)	Jul-2004
EU857022	FluA_H3N2_HA	A/Hong Kong/CUHK50552/1998(H3N2)	Dec-1998
EU857023	FluA_H3N2_HA	A/Hong Kong/CUHK50563/2004(H3N2)	Jul-2004
EU857024	FluA_H3N2_HA	A/Hong Kong/CUHK50600/2004(H3N2)	Jul-2004
EU857025	FluA_H3N2_HA	A/Hong Kong/CUHK50722/1998(H3N2)	Dec-1998

GenBank accession no.	Type	Isolate ID	Collection date
EU857026	FluA_H3N2_HA	A/Hong Kong/CUHK50895/2003(H3N2)	Jul-2003
EU857027	FluA_H3N2_HA	A/Hong Kong/CUHK50952/2003(H3N2)	Jul-2003
EU857028	FluA_H3N2_HA	A/Hong Kong/CUHK5111/2003(H3N2)	Jan-2003
EU857029	FluA_H3N2_HA	A/Hong Kong/CUHK51353/2003(H3N2)	Jul-2003
EU857030	FluA_H3N2_HA	A/Hong Kong/CUHK51380/2003(H3N2)	Jul-2003
EU857031	FluA_H3N2_HA	A/Hong Kong/CUHK51424/2001(H3N2)	Dec-2001
EU857032	FluA_H3N2_HA	A/Hong Kong/CUHK51431/2001(H3N2)	Dec-2001
EU857033	FluA_H3N2_HA	A/Hong Kong/CUHK51490/2001(H3N2)	Dec-2001
EU857035	FluA_H3N2_HA	A/Hong Kong/CUHK52011/2003(H3N2)	Aug-2003
EU857036	FluA_H3N2_HA	A/Hong Kong/CUHK52247/2000(H3N2)	Dec-2000
EU857037	FluA_H3N2_HA	A/Hong Kong/CUHK52274/2004(H3N2)	Aug-2004
EU857038	FluA_H3N2_HA	A/Hong Kong/CUHK52390/2004(H3N2)	Aug-2004
EU857039	FluA_H3N2_HA	A/Hong Kong/CUHK52467/2003(H3N2)	Aug-2003
EU857040	FluA_H3N2_HA	A/Hong Kong/CUHK5250/2002(H3N2)	Jan-2002
EU857041	FluA_H3N2_HA	A/Hong Kong/CUHK5251/2002(H3N2)	Jan-2002
EU857044	FluA_H3N2_HA	A/Hong Kong/CUHK52923/2003(H3N2)	Aug-2003
EU857045	FluA_H3N2_HA	A/Hong Kong/CUHK52941/2004(H3N2)	Aug-2004
EU857046	FluA_H3N2_HA	A/Hong Kong/CUHK5296/2002(H3N2)	Jan-2002
EU857047	FluA_H3N2_HA	A/Hong Kong/CUHK5300/2005(H3N2)	Jan-2005
EU857048	FluA_H3N2_HA	A/Hong Kong/CUHK53004/2006(H3N2)	Mar-2006
EU857050	FluA_H3N2_HA	A/Hong Kong/CUHK53123/2002(H3N2)	Dec-2002
EU857052	FluA_H3N2_HA	A/Hong Kong/CUHK53327/2002(H3N2)	Dec-2002
EU857053	FluA_H3N2_HA	A/Hong Kong/CUHK53726/2004(H3N2)	Sep-2004
EU857054	FluA_H3N2_HA	A/Hong Kong/CUHK53732/2004(H3N2)	Sep-2004
EU857055	FluA_H3N2_HA	A/Hong Kong/CUHK53766/2004(H3N2)	Sep-2004
EU857057	FluA_H3N2_HA	A/Hong Kong/CUHK53907/2006(H3N2)	Mar-2006
EU857058	FluA_H3N2_HA	A/Hong Kong/CUHK5567/2005(H3N2)	Jan-2005
EU857059	FluA_H3N2_HA	A/Hong Kong/CUHK5627/2003(H3N2)	Jan-2003
EU857061	FluA_H3N2_HA	A/Hong Kong/CUHK5723/2003(H3N2)	Jan-2003
EU857062	FluA_H3N2_HA	A/Hong Kong/CUHK60052/2006(H3N2)	Jun-2006
EU857063	FluA_H3N2_HA	A/Hong Kong/CUHK6205/2003(H3N2)	Feb-2003
EU857064	FluA_H3N2_HA	A/Hong Kong/CUHK6206/2002(H3N2)	Feb-2002
EU857066	FluA_H3N2_HA	A/Hong Kong/CUHK6262/2003(H3N2)	Feb-2003
EU857067	FluA_H3N2_HA	A/Hong Kong/CUHK6377/2003(H3N2)	Feb-2003
EU857068	FluA_H3N2_HA	A/Hong Kong/CUHK6383/2003(H3N2)	Feb-2003
EU857070	FluA_H3N2_HA	A/Hong Kong/CUHK64082/2006(H3N2)	Sep-2006
EU857071	FluA_H3N2_HA	A/Hong Kong/CUHK6422/2005(H3N2)	Jan-2005
EU857072	FluA_H3N2_HA	A/Hong Kong/CUHK6427/2003(H3N2)	Feb-2003
EU857074	FluA_H3N2_HA	A/Hong Kong/CUHK65241/2006(H3N2)	Sep-2006
EU857076	FluA_H3N2_HA	A/Hong Kong/CUHK66001/2006(H3N2)	Oct-2006
EU857078	FluA_H3N2_HA	A/Hong Kong/CUHK68792/2006(H3N2)	Dec-2006
EU857079	FluA_H3N2_HA	A/Hong Kong/CUHK6898/2005(H3N2)	Feb-2005
EU857080	FluA_H3N2_HA	A/Hong Kong/CUHK69904/2006(H3N2)	Dec-2006
EU857081	FluA_H3N2_HA	A/Hong Kong/CUHK70435/2003(H3N2)	Sep-2003
EU857082	FluA_H3N2_HA	A/Hong Kong/CUHK7047/2005(H3N2)	Feb-2005
EU857083	FluA_H3N2_HA	A/Hong Kong/CUHK71313/2003(H3N2)	Sep-2003
EU857084	FluA_H3N2_HA	A/Hong Kong/CUHK72014/2003(H3N2)	Sep-2003
EU857085	FluA_H3N2_HA	A/Hong Kong/CUHK7221/2005(H3N2)	Feb-2005
EU857086	FluA_H3N2_HA	A/Hong Kong/CUHK74438/2003(H3N2)	Nov-2003
EU857087	FluA_H3N2_HA	A/Hong Kong/CUHK74457/2003(H3N2)	Nov-2003
EU857088	FluA_H3N2_HA	A/Hong Kong/CUHK7546/2005(H3N2)	Feb-2005
EU857089	FluA_H3N2_HA	A/Hong Kong/CUHK75994/2003(H3N2)	Dec-2003
EU857090	FluA_H3N2_HA	A/Hong Kong/CUHK7670/2005(H3N2)	Mar-2005
EU857093	FluA_H3N2_HA	A/Hong Kong/CUHK8333/2005(H3N2)	Mar-2005
EU857094	FluA_H3N2_HA	A/Hong Kong/CUHK83422/2003(H3N2)	Dec-2003
MF955212	FluA_H3N2_HA	A/Hong Kong/CUHK27868/2011(H3N2)	Sep-2011
MF955213	FluA_H3N2_HA	A/Hong Kong/CUHK59603/2009(H3N2)	Apr-2009
MF955214	FluA_H3N2_HA	A/Hong Kong/CUHK41162/2010(H3N2)	Sep-2010
MF955215	FluA_H3N2_HA	A/Hong Kong/CUHK18263/1996(H3N2)	Jun-1996
MF955216	FluA_H3N2_HA	A/Hong Kong/CUHK66043/2008(H3N2)	Apr-2008
MF955217	FluA_H3N2_HA	A/Hong Kong/CUHK42826/2007(H3N2)	Sep-2007
MF955218	FluA_H3N2_HA	A/Hong Kong/CUHK32398/2011(H3N2)	Dec-2011
MF955219	FluA_H3N2_HA	A/Hong Kong/CUHK03421/1996(H3N2)	Mar-1996
MF955220	FluA_H3N2_HA	A/Hong Kong/CUHK43773/2012(H3N2)	Sep-2012
MF955221	FluA_H3N2_HA	A/Hong Kong/CUHK49134/2012(H3N2)	Dec-2012
MF955222	FluA_H3N2_HA	A/Hong Kong/CUHK74487/2008(H3N2)	Aug-2008
MF955223	FluA_H3N2_HA	A/Hong Kong/CUHK10703/1996(H3N2)	Mar-1996
MF955224	FluA_H3N2_HA	A/Hong Kong/CUHK28267/2011(H3N2)	Sep-2011
MF955225	FluA_H3N2_HA	A/Hong Kong/CUHK03367/1996(H3N2)	Mar-1996

GenBank accession no.	Type	Isolate ID	Collection date
MF955226	FluA_H3N2_HA	A/Hong Kong/CUHK68950/2008(H3N2)	May-2008
MF955227	FluA_H3N2_HA	A/Hong Kong/CUHK70724/2009(H3N2)	Apr-2009
MF955228	FluA_H3N2_HA	A/Hong Kong/CUHK40360/2010(H3N2)	Sep-2010
MF955229	FluA_H3N2_HA	A/Hong Kong/CUHK41243/2012(H3N2)	Aug-2012
MF955230	FluA_H3N2_HA	A/Hong Kong/CUHK35729/2012(H3N2)	May-2012
MF955231	FluA_H3N2_HA	A/Hong Kong/CUHK21764/1996(H3N2)	Jun-1996
MF955232	FluA_H3N2_HA	A/Hong Kong/CUHK17970/1996(H3N2)	May-1996
MF955233	FluA_H3N2_HA	A/Hong Kong/CUHK41913/2012(H3N2)	Aug-2012
MF955234	FluA_H3N2_HA	A/Hong Kong/CUHK47352/2012(H3N2)	Nov-2012
MF955235	FluA_H3N2_HA	A/Hong Kong/CUHK62335/2008(H3N2)	Feb-2008
MF955236	FluA_H3N2_HA	A/Hong Kong/CUHK65310/2008(H3N2)	Mar-2008
MF955237	FluA_H3N2_HA	A/Hong Kong/CUHK72035/2008(H3N2)	Jul-2008
MF955238	FluA_H3N2_HA	A/Hong Kong/CUHK61355/2007(H3N2)	Jan-2007
MF955239	FluA_H3N2_HA	A/Hong Kong/CUHK62203/2007(H3N2)	Feb-2007
MF955240	FluA_H3N2_HA	A/Hong Kong/CUHK62842/2007(H3N2)	Feb-2007
MF955241	FluA_H3N2_HA	A/Hong Kong/CUHK35004/2012(H3N2)	May-2012
MF955242	FluA_H3N2_HA	A/Hong Kong/CUHK72058/2009(H3N2)	May-2009
MF955243	FluA_H3N2_HA	A/Hong Kong/CUHK39813/2010(H3N2)	Aug-2010
MF955244	FluA_H3N2_HA	A/Hong Kong/CUHK71449/2009(H3N2)	Apr-2009
MF955245	FluA_H3N2_HA	A/Hong Kong/CUHK77585/2008(H3N2)	Sep-2008
MF955246	FluA_H3N2_HA	A/Hong Kong/CUHK66598/2009(H3N2)	Jan-2009
MF955247	FluA_H3N2_HA	A/Hong Kong/CUHK43402/2010(H3N2)	Oct-2010
MF955248	FluA_H3N2_HA	A/Hong Kong/CUHK27565/2012(H3N2)	Feb-2012
MF955249	FluA_H3N2_HA	A/Hong Kong/CUHK33530/2011(H3N2)	Dec-2011
MF955250	FluA_H3N2_HA	A/Hong Kong/CUHK26783/2011(H3N2)	Aug-2011
MF955251	FluA_H3N2_HA	A/Hong Kong/CUHK33163/2011(H3N2)	Dec-2011
MF955252	FluA_H3N2_HA	A/Hong Kong/CUHK66798/2007(H3N2)	Apr-2007
MF955253	FluA_H3N2_HA	A/Hong Kong/CUHK64331/2007(H3N2)	Mar-2007
MF955254	FluA_H3N2_HA	A/Hong Kong/CUHK69991/2007(H3N2)	Jun-2007
MF955255	FluA_H3N2_HA	A/Hong Kong/CUHK74215/2008(H3N2)	Aug-2008
MF955256	FluA_H3N2_HA	A/Hong Kong/CUHK73440/2008(H3N2)	Jul-2008
MF955257	FluA_H3N2_HA	A/Hong Kong/CUHK70458/2008(H3N2)	Jun-2008
MF955258	FluA_H3N2_HA	A/Hong Kong/CUHK72215/2008(H3N2)	Jul-2008
MF955259	FluA_H3N2_HA	A/Hong Kong/CUHK39450/2012(H3N2)	Jul-2012
MF955260	FluA_H3N2_HA	A/Hong Kong/CUHK45712/2012(H3N2)	Oct-2012
MF955261	FluA_H3N2_HA	A/Hong Kong/CUHK15232/1996(H3N2)	Apr-1996
MF955262	FluA_H3N2_HA	A/Hong Kong/CUHK44756/2010(H3N2)	Nov-2010
MF955263	FluA_H3N2_HA	A/Hong Kong/CUHK46938/2010(H3N2)	Dec-2010
MF955264	FluA_H3N2_HA	A/Hong Kong/CUHK32113/2011(H3N2)	Dec-2011
MF955265	FluA_H3N2_HA	A/Hong Kong/CUHK15308/1996(H3N2)	Apr-1996
MF955266	FluA_H3N2_HA	A/Hong Kong/CUHK21932/1996(H3N2)	Jun-1996
MF955267	FluA_H3N2_HA	A/Hong Kong/CUHK29206/1996(H3N2)	Sep-1996
MF955268	FluA_H3N2_HA	A/Hong Kong/CUHK72075/2009(H3N2)	May-2009
MF955269	FluA_H3N2_HA	A/Hong Kong/CUHK73167/2009(H3N2)	May-2009
MF955270	FluA_H3N2_HA	A/Hong Kong/CUHK40227/2010(H3N2)	Sep-2010
MF955271	FluA_H3N2_HA	A/Hong Kong/CUHK80068/2008(H3N2)	Nov-2008
MF955272	FluA_H3N2_HA	A/Hong Kong/CUHK59944/2009(H3N2)	May-2009
MF955273	FluA_H3N2_HA	A/Hong Kong/CUHK67251/2009(H3N2)	Jan-2009
MF955274	FluA_H3N2_HA	A/Hong Kong/CUHK25423/2011(H3N2)	Jul-2011
MF955275	FluA_H3N2_HA	A/Hong Kong/CUHK30771/2011(H3N2)	Nov-2011
MF955276	FluA_H3N2_HA	A/Hong Kong/CUHK31374/2011(H3N2)	Nov-2011
MF955277	FluA_H3N2_HA	A/Hong Kong/CUHK40759/2010(H3N2)	Sep-2010
MF955278	FluA_H3N2_HA	A/Hong Kong/CUHK44339/2010(H3N2)	Nov-2010
MF955279	FluA_H3N2_HA	A/Hong Kong/CUHK23961/2011(H3N2)	Jun-2011
MF955280	FluA_H3N2_HA	A/Hong Kong/CUHK67413/2008(H3N2)	Apr-2008
MF955281	FluA_H3N2_HA	A/Hong Kong/CUHK60258/2007(H3N2)	Jan-2007
MF955282	FluA_H3N2_HA	A/Hong Kong/CUHK62981/2007(H3N2)	Feb-2007
MF955283	FluA_H3N2_HA	A/Hong Kong/CUHK72228/2007(H3N2)	Jul-2007
MF955284	FluA_H3N2_HA	A/Hong Kong/CUHK73588/2007(H3N2)	Aug-2007
MF955285	FluA_H3N2_HA	A/Hong Kong/CUHK66092/2008(H3N2)	Apr-2008
MF955286	FluA_H3N2_HA	A/Hong Kong/CUHK63130/2007(H3N2)	Feb-2007
MF955287	FluA_H3N2_HA	A/Hong Kong/CUHK66156/2007(H3N2)	Apr-2007
MF955288	FluA_H3N2_HA	A/Hong Kong/CUHK71638/2007(H3N2)	Jul-2007
MF955289	FluA_H3N2_HA	A/Hong Kong/CUHK41897/2010(H3N2)	Sep-2010
MF955290	FluA_H3N2_HA	A/Hong Kong/CUHK15154/1996(H3N2)	Apr-1996
MF955291	FluA_H3N2_HA	A/Hong Kong/CUHK21907/1996(H3N2)	Jun-1996
MF955292	FluA_H3N2_HA	A/Hong Kong/CUHK21971/1996(H3N2)	Jan-1996
MF955293	FluA_H3N2_HA	A/Hong Kong/CUHK03269/1996(H3N2)	Mar-1996
MF955294	FluA_H3N2_HA	A/Hong Kong/CUHK38327/2010(H3N2)	Jul-2010

GenBank accession no.	Type	Isolate ID	Collection date
MF955295	FluA_H3N2_HA	A/Hong Kong/CUHK42094/2010(H3N2)	Sep-2010
MF955296	FluA_H3N2_HA	A/Hong Kong/CUHK20677/2011(H3N2)	Apr-2011
MF955297	FluA_H3N2_HA	A/Hong Kong/CUHK72474/2008(H3N2)	Jul-2008
MF955298	FluA_H3N2_HA	A/Hong Kong/CUHK69295/2009(H3N2)	Mar-2009
MF955299	FluA_H3N2_HA	A/Hong Kong/CUHK36548/2010(H3N2)	Jun-2010
MF955300	FluA_H3N2_HA	A/Hong Kong/CUHK30631/2012(H3N2)	Mar-2012
MF955301	FluA_H3N2_HA	A/Hong Kong/CUHK40597/2012(H3N2)	Jul-2012
MF955302	FluA_H3N2_HA	A/Hong Kong/CUHK41586/2012(H3N2)	Aug-2012
MF955303	FluA_H3N2_HA	A/Hong Kong/CUHK23993/2011(H3N2)	Jun-2011
MF955304	FluA_H3N2_HA	A/Hong Kong/CUHK29342/2011(H3N2)	Oct-2011
MF955305	FluA_H3N2_HA	A/Hong Kong/CUHK28838/2012(H3N2)	Feb-2012
MF955306	FluA_H3N2_HA	A/Hong Kong/CUHK72836/2008(H3N2)	Jul-2008
MF955307	FluA_H3N2_HA	A/Hong Kong/CUHK74704/2008(H3N2)	Aug-2008
MF955308	FluA_H3N2_HA	A/Hong Kong/CUHK67380/2009(H3N2)	Jan-2009
MF955309	FluA_H3N2_HA	A/Hong Kong/CUHK65050/2007(H3N2)	Mar-2007
MF955310	FluA_H3N2_HA	A/Hong Kong/CUHK72318/2008(H3N2)	Jul-2008
MF955311	FluA_H3N2_HA	A/Hong Kong/CUHK39295/2010(H3N2)	Aug-2010
MF955312	FluA_H3N2_HA	A/Hong Kong/CUHK39459/2010(H3N2)	Aug-2010
MF955313	FluA_H3N2_HA	A/Hong Kong/CUHK41743/2010(H3N2)	Sep-2010
MF955314	FluA_H3N2_HA	A/Hong Kong/CUHK68016/2009(H3N2)	Feb-2009
MF955315	FluA_H3N2_HA	A/Hong Kong/CUHK73723/2009(H3N2)	Jun-2009
MF955316	FluA_H3N2_HA	A/Hong Kong/CUHK36649/2010(H3N2)	Jun-2010
MF955317	FluA_H3N2_HA	A/Hong Kong/CUHK14229/2011(H3N2)	Jan-2011
MF955318	FluA_H3N2_HA	A/Hong Kong/CUHK16933/2011(H3N2)	Feb-2011
MF955319	FluA_H3N2_HA	A/Hong Kong/CUHK29096/2011(H3N2)	Oct-2011
MF955320	FluA_H3N2_HA	A/Hong Kong/CUHK73629/2009(H3N2)	Jun-2009
MF955321	FluA_H3N2_HA	A/Hong Kong/CUHK38606/2010(H3N2)	Aug-2010
MF955322	FluA_H3N2_HA	A/Hong Kong/CUHK39699/2010(H3N2)	Aug-2010
MF955323	FluA_H3N2_HA	A/Hong Kong/CUHK28253/2012(H3N2)	Feb-2012
MF955324	FluA_H3N2_HA	A/Hong Kong/CUHK33681/2012(H3N2)	Apr-2012
MF955325	FluA_H3N2_HA	A/Hong Kong/CUHK40215/2012(H3N2)	Jul-2012
MF955326	FluA_H3N2_HA	A/Hong Kong/CUHK31702/2011(H3N2)	Nov-2011
MF955327	FluA_H3N2_HA	A/Hong Kong/CUHK31982/2011(H3N2)	Nov-2011
MF955328	FluA_H3N2_HA	A/Hong Kong/CUHK26268/2012(H3N2)	Jan-2012
MF955329	FluA_H3N2_HA	A/Hong Kong/CUHK72079/2009(H3N2)	May-2009
MF955330	FluA_H3N2_HA	A/Hong Kong/CUHK63441/2007(H3N2)	Mar-2007
MF955331	FluA_H3N2_HA	A/Hong Kong/CUHK78641/2008(H3N2)	Oct-2008
MF955332	FluA_H3N2_HA	A/Hong Kong/CUHK67726/2009(H3N2)	Feb-2009
MF955333	FluA_H3N2_HA	A/Hong Kong/CUHK70258/2009(H3N2)	Mar-2009
MF955334	FluA_H3N2_HA	A/Hong Kong/CUHK63676/2007(H3N2)	Mar-2007
MF955335	FluA_H3N2_HA	A/Hong Kong/CUHK71881/2008(H3N2)	Jul-2008
MF955336	FluA_H3N2_HA	A/Hong Kong/CUHK73144/2008(H3N2)	Jul-2008
MF955337	FluA_H3N2_HA	A/Hong Kong/CUHK18334/1996(H3N2)	Jun-1996
MF955338	FluA_H3N2_HA	A/Hong Kong/CUHK10886/1996(H3N2)	Apr-1996
MF955339	FluA_H3N2_HA	A/Hong Kong/CUHK29537/2011(H3N2)	Oct-2011
MF955340	FluA_H3N2_HA	A/Hong Kong/CUHK29782/2011(H3N2)	Oct-2011
MF955341	FluA_H3N2_HA	A/Hong Kong/CUHK06513/2012(H3N2)	Jan-2012
MF955342	FluA_H3N2_HA	A/Hong Kong/CUHK24671/2011(H3N2)	Jul-2011
MF955343	FluA_H3N2_HA	A/Hong Kong/CUHK28795/2011(H3N2)	Sep-2011
MF955344	FluA_H3N2_HA	A/Hong Kong/CUHK28994/2011(H3N2)	Oct-2011
MF955345	FluA_H3N2_HA	A/Hong Kong/CUHK48508/2012(H3N2)	Dec-2012
MF955346	FluA_H3N2_HA	A/Hong Kong/CUHK10954/1996(H3N2)	Apr-1996
MF955347	FluA_H3N2_HA	A/Hong Kong/CUHK18325/1996(H3N2)	Jun-1996
MF955348	FluA_H3N2_HA	A/Hong Kong/CUHK26560/2012(H3N2)	Jan-2012
MF955349	FluA_H3N2_HA	A/Hong Kong/CUHK37322/2012(H3N2)	Jun-2012
MF955350	FluA_H3N2_HA	A/Hong Kong/CUHK38000/2012(H3N2)	Jun-2012
MF955351	FluA_H3N2_HA	A/Hong Kong/CUHK45722/2010(H3N2)	Dec-2010
MF955352	FluA_H3N2_HA	A/Hong Kong/CUHK69107/2007(H3N2)	Jun-2007
MF955353	FluA_H3N2_HA	A/Hong Kong/CUHK60906/2008(H3N2)	Jan-2008
MF955354	FluA_H3N2_HA	A/Hong Kong/CUHK77209/2008(H3N2)	Aug-2008
MF955355	FluA_H3N2_HA	A/Hong Kong/CUHK65249/2007(H3N2)	Mar-2007
MF955356	FluA_H3N2_HA	A/Hong Kong/CUHK68205/2007(H3N2)	May-2007
MF955357	FluA_H3N2_HA	A/Hong Kong/CUHK41034/2010(H3N2)	Sep-2010
MF955358	FluA_H3N2_HA	A/Hong Kong/CUHK42370/2010(H3N2)	Oct-2010
MF955359	FluA_H3N2_HA	A/Hong Kong/CUHK42691/2010(H3N2)	Oct-2010
MF955360	FluA_H3N2_HA	A/Hong Kong/CUHK66835/2009(H3N2)	Jan-2009
MF955361	FluA_H3N2_HA	A/Hong Kong/CUHK37893/2010(H3N2)	Jul-2010
MF955362	FluA_H3N2_HA	A/Hong Kong/CUHK39965/2010(H3N2)	Aug-2010
MF955363	FluA_H3N2_HA	A/Hong Kong/CUHK32581/2012(H3N2)	Apr-2012

GenBank accession no.	Type	Isolate ID	Collection date
MF955364	FluA_H3N2_HA	A/Hong Kong/CUHK10821/1996(H3N2)	Apr-1996
MF955365	FluA_H3N2_HA	A/Hong Kong/CUHK30085/2012(H3N2)	Mar-2012
MF955366	FluA_H3N2_HA	A/Hong Kong/CUHK33255/2012(H3N2)	Apr-2012
MF955367	FluA_H3N2_HA	A/Hong Kong/CUHK34445/2012(H3N2)	May-2012
MF955368	FluA_H3N2_HA	A/Hong Kong/CUHK71868/2009(H3N2)	Apr-2009
MF955369	FluA_H3N2_HA	A/Hong Kong/CUHK38864/2010(H3N2)	Aug-2010
MF955370	FluA_H3N2_HA	A/Hong Kong/CUHK40637/2010(H3N2)	Sep-2010
MF955371	FluA_H3N2_HA	A/Hong Kong/CUHK15321/1996(H3N2)	May-1996
MF955372	FluA_H3N2_HA	A/Hong Kong/CUHK15361/1996(H3N2)	May-1996
MF955373	FluA_H3N2_HA	A/Hong Kong/CUHK18255/1996(H3N2)	Jun-1996
MF955374	FluA_H3N2_HA	A/Hong Kong/CUHK43137/2012(H3N2)	Sep-2012
MF955375	FluA_H3N2_HA	A/Hong Kong/CUHK10905/1996(H3N2)	Apr-1996
MF955376	FluA_H3N2_HA	A/Hong Kong/CUHK10930/1996(H3N2)	Apr-1996
MF955377	FluA_H3N2_HA	A/Hong Kong/CUHK70905/2009(H3N2)	Apr-2009
MF955378	FluA_H3N2_HA	A/Hong Kong/CUHK70481/2007(H3N2)	Jun-2007
MF955379	FluA_H3N2_HA	A/Hong Kong/CUHK70672/2007(H3N2)	Jun-2007
MF955380	FluA_H3N2_HA	A/Hong Kong/CUHK71333/2007(H3N2)	Jul-2007
MF955381	FluA_H3N2_HA	A/Hong Kong/CUHK67465/2007(H3N2)	May-2007
MF955382	FluA_H3N2_HA	A/Hong Kong/CUHK70137/2007(H3N2)	Jun-2007
MF955383	FluA_H3N2_HA	A/Hong Kong/CUHK66547/2009(H3N2)	Jan-2009
MF955384	FluA_H3N2_HA	A/Hong Kong/CUHK68649/2009(H3N2)	Feb-2009
MF955385	FluA_H3N2_HA	A/Hong Kong/CUHK70803/2009(H3N2)	Apr-2009
MF955386	FluA_H3N2_HA	A/Hong Kong/CUHK64533/2008(H3N2)	Mar-2008
MF955387	FluA_H3N2_HA	A/Hong Kong/CUHK73085/2008(H3N2)	Jul-2008
MF955388	FluA_H3N2_HA	A/Hong Kong/CUHK82526/2008(H3N2)	Dec-2008
MF955389	FluA_H3N2_HA	A/Hong Kong/CUHK13623/2011(H3N2)	Jan-2011
MF955390	FluA_H3N2_HA	A/Hong Kong/CUHK10992/1996(H3N2)	Apr-1996
MF955391	FluA_H3N2_HA	A/Hong Kong/CUHK15208/1996(H3N2)	Apr-1996
MF955392	FluA_H3N2_HA	A/Hong Kong/CUHK26553/1996(H3N2)	Aug-1996
MF955393	FluA_H3N2_HA	A/Hong Kong/CUHK10715/1996(H3N2)	Mar-1996
MF955394	FluA_H3N2_HA	A/Hong Kong/CUHK10922/1996(H3N2)	Apr-1996
MF955395	FluA_H3N2_HA	A/Hong Kong/CUHK10970/1996(H3N2)	Apr-1996
MF955396	FluA_H3N2_HA	A/Hong Kong/CUHK64597/2007(H3N2)	Mar-2007
MF955397	FluA_H3N2_HA	A/Hong Kong/CUHK71129/2007(H3N2)	Jun-2007
MF955398	FluA_H3N2_HA	A/Hong Kong/CUHK72743/2007(H3N2)	Jul-2007
MF955399	FluA_H3N2_HA	A/Hong Kong/CUHK62440/2007(H3N2)	Feb-2007
MF955400	FluA_H3N2_HA	A/Hong Kong/CUHK62789/2007(H3N2)	Feb-2007
MF955401	FluA_H3N2_HA	A/Hong Kong/CUHK63295/2007(H3N2)	Feb-2007
MF955402	FluA_H3N2_HA	A/Hong Kong/CUHK73649/2009(H3N2)	Jun-2009
MF955403	FluA_H3N2_HA	A/Hong Kong/CUHK40492/2010(H3N2)	Sep-2010
MF955404	FluA_H3N2_HA	A/Hong Kong/CUHK43136/2010(H3N2)	Oct-2010
MF955405	FluA_H3N2_HA	A/Hong Kong/CUHK72991/2008(H3N2)	Jul-2008
MF955406	FluA_H3N2_HA	A/Hong Kong/CUHK68236/2009(H3N2)	Feb-2009
MF955407	FluA_H3N2_HA	A/Hong Kong/CUHK73480/2009(H3N2)	Jun-2009
MF955408	FluA_H3N2_HA	A/Hong Kong/CUHK61864/2007(H3N2)	Feb-2007
MF955409	FluB_HA	B/Hong Kong/CUHK954/1997	Feb-1997
MF955410	FluB_HA	B/Hong Kong/CUHK5911/2002	Jan-2002
MF955411	FluB_HA	B/Hong Kong/CUHK65229/2007	Mar-2007
MF955412	FluB_HA	B/Hong Kong/CUHK47908/2012	Nov-2012
MF955413	FluB_HA	B/Hong Kong/CUHK33652/2011	Dec-2011
MF955414	FluB_HA	B/Hong Kong/CUHK15535/1998	Jun-1998
MF955415	FluB_HA	B/Hong Kong/CUHK64639/2008	Mar-2008
MF955416	FluB_HA	B/Hong Kong/CUHK10649/1996	Mar-1996
MF955417	FluB_HA	B/Hong Kong/CUHK27358/2010	Feb-2010
MF955418	FluB_HA	B/Hong Kong/CUHK50687/2004	Jul-2004
MF955419	FluB_HA	B/Hong Kong/CUHK74303/2007	Aug-2007
MF955420	FluB_HA	B/Hong Kong/CUHK13967 /1999	May-1999
MF955421	FluB_HA	B/Hong Kong/CUHK33668/2012	Apr-2012
MF955422	FluB_HA	B/Hong Kong/CUHK33761/2012	Apr-2012
MF955423	FluB_HA	B/Hong Kong/CUHK21897/1996	Jun-1996
MF955424	FluB_HA	B/Hong Kong/CUHK15517 /1998	Jun-1998
MF955425	FluB_HA	B/Hong Kong/CUHK50325/2002	Oct-2002
MF955426	FluB_HA	B/Hong Kong/CUHK11835/1999	Mar-1999
MF955427	FluB_HA	B/Hong Kong/CUHK39404/2010	Aug-2010
MF955428	FluB_HA	B/Hong Kong/CUHK46712/2010	Dec-2010
MF955429	FluB_HA	B/Hong Kong/CUHK45711/2000	Nov-2000
MF955430	FluB_HA	B/Hong Kong/CUHK65888/2008	Apr-2008
MF955431	FluB_HA	B/Hong Kong/CUHK16551/1998	Jul-1998
MF955432	FluB_HA	B/Hong Kong/CUHK62733/2008	Feb-2008

GenBank accession no.	Type	Isolate ID	Collection date
MF955433	FluB_HA	B/Hong Kong/CUHK11995/1999	Mar-1999
MF955434	FluB_HA	B/Hong Kong/CUHK63933/2007	Mar-2007
MF955435	FluB_HA	B/Hong Kong/CUHK29315/2012	Mar-2012
MF955436	FluB_HA	B/Hong Kong/CUHK46202/2010	Dec-2010
MF955437	FluB_HA	B/Hong Kong/CUHK16087/1998	Jun-1998
MF955438	FluB_HA	B/Hong Kong/CUHK16854/1998	Jul-1998
MF955439	FluB_HA	B/Hong Kong/CUHK15855/1998	Jun-1998
MF955440	FluB_HA	B/Hong Kong/CUHK45550/2000	Nov-2000
MF955441	FluB_HA	B/Hong Kong/CUHK052533/2000	Dec-2000
MF955442	FluB_HA	B/Hong Kong/CUHK6030/2001	Feb-2001
MF955443	FluB_HA	B/Hong Kong/CUHK30662/2011	Nov-2011
MF955444	FluB_HA	B/Hong Kong/CUHK51180/2002	Oct-2002
MF955445	FluB_HA	B/Hong Kong/CUHK18357/1996	Jun-1996
MF955446	FluB_HA	B/Hong Kong/CUHK460/1997	Jan-1997
MF955447	FluB_HA	B/Hong Kong/CUHK17239/1998	Jul-1998
MF955448	FluB_HA	B/Hong Kong/CUHK16953/1998	Jul-1998
MF955449	FluB_HA	B/Hong Kong/CUHK11868/1999	Mar-1999
MF955450	FluB_HA	B/Hong Kong/CUHK5724/2003	Jan-2003
MF955451	FluB_HA	B/Hong Kong/CUHK51404/2004	Jul-2004
MF955452	FluB_HA	B/Hong Kong/CUHK9350/2005	Mar-2005
MF955453	FluB_HA	B/Hong Kong/CUHK42989 /2007	Sep-2007
MF955454	FluB_HA	B/Hong Kong/CUHK24560/2000	May-2000
MF955455	FluB_HA	B/Hong Kong/CUHK22392/2000	Mar-2000
MF955456	FluB_HA	B/Hong Kong/CUHK52005/2000	Dec-2000
MF955457	FluB_HA	B/Hong Kong/CUHK12003 /1999	Mar-1999
MF955458	FluB_HA	B/Hong Kong/CUHK14124/1999	May-1999
MF955459	FluB_HA	B/Hong Kong/CUHK23174/2000	Mar-2000
MF955460	FluB_HA	B/Hong Kong/CUHK22465/2001	May-2001
MF955461	FluB_HA	B/Hong Kong/CUHK51178/2002	Oct-2002
MF955462	FluB_HA	B/Hong Kong/CUHK22741/2001	May-2001
MF955463	FluB_HA	B/Hong Kong/CUHK23356/2000	Apr-2000
MF955464	FluB_HA	B/Hong Kong/CUHK6611/2003	Feb-2003
MF955465	FluB_HA	B/Hong Kong/CUHK21447/2003	Mar-2003
MF955466	FluB_HA	B/Hong Kong/CUHK52370/2004	Aug-2004
MF955467	FluB_HA	B/Hong Kong/CUHK13481/2003	Mar-2003
MF955468	FluB_HA	B/Hong Kong/CUHK16575/1998	Jul-1998
MF955469	FluB_HA	B/Hong Kong/CUHK17605/1998	Aug-1998
MF955470	FluB_HA	B/Hong Kong/CUHK26127/1996	Jul-1996
MF955471	FluB_HA	B/Hong Kong/CUHK4120/1997	Mar-1997
MF955472	FluB_HA	B/Hong Kong/CUHK29309 /2000	Oct-2000
MF955473	FluB_HA	B/Hong Kong/CUHK14186/1999	May-1999
MF955474	FluB_HA	B/Hong Kong/CUHK22706/2000	Feb-2000
MF955475	FluB_HA	B/Hong Kong/CUHK23143/2011	Jun-2011
MF955476	FluB_HA	B/Hong Kong/CUHK31852/2011	Nov-2011
MF955477	FluB_HA	B/Hong Kong/CUHK14156/2011	Jan-2011
MF955478	FluB_HA	B/Hong Kong/CUHK43211/2010	Oct-2010
MF955479	FluB_HA	B/Hong Kong/CUHK4348/1997	Mar-1997
MF955480	FluB_HA	B/Hong Kong/CUHK16076/1998	Jun-1998
MF955481	FluB_HA	B/Hong Kong/CUHK10823/1996	Apr-1996
MF955482	FluB_HA	B/Hong Kong/CUHK10549/1996	Mar-1996
MF955483	FluB_HA	B/Hong Kong/CUHK18268/1996	Jun-1996
MF955484	FluB_HA	B/Hong Kong/CUHK33955/2010	May-2010
MF955485	FluB_HA	B/Hong Kong/CUHK12338/2005	May-2005
MF955486	FluB_HA	B/Hong Kong/CUHK26813/2010	Jan-2010
MF955487	FluB_HA	B/Hong Kong/CUHK66047/2008	Apr-2008
MF955488	FluB_HA	B/Hong Kong/CUHK63322/2008	Feb-2008
MF955489	FluB_HA	B/Hong Kong/CUHK64419/2008	Mar-2008
MF955490	FluB_HA	B/Hong Kong/CUHK32498 /2012	Apr-2012
MF955491	FluB_HA	B/Hong Kong/CUHK33099/2012	Apr-2012
MF955492	FluB_HA	B/Hong Kong/CUHK30032/2012	Mar-2012
MF955493	FluB_HA	B/Hong Kong/CUHK23377/2011	Jun-2011
MF955494	FluB_HA	B/Hong Kong/CUHK29189/2012	Feb-2012
MF955495	FluB_HA	B/Hong Kong/CUHK33106/2012	Apr-2012
MF955496	FluB_HA	B/Hong Kong/CUHK5806/2001	Jan-2001
MF955497	FluB_HA	B/Hong Kong/CUHK11672/1999	Feb-1999
MF955498	FluB_HA	B/Hong Kong/CUHK10656 /1996	Mar-1996
MF955499	FluB_HA	B/Hong Kong/CUHK16364/1998	Jul-1998
MF955500	FluB_HA	B/Hong Kong/CUHK21441/2011	May-2011
MF955501	FluB_HA	B/Hong Kong/CUHK63615/2008	Mar-2008

GenBank accession no.	Type	Isolate ID	Collection date
MF955502	FluB_HA	B/Hong Kong/CUHK42491 /2007	Sep-2007
MF955503	FluB_HA	B/Hong Kong/CUHK46173/2007	Nov-2007
MF955504	FluB_HA	B/Hong Kong/CUHK66994/2007	Apr-2007
MF955505	FluB_HA	B/Hong Kong/CUHK64081/2008	Mar-2008
MF955506	FluB_HA	B/Hong Kong/CUHK18490/2011	Mar-2011
MF955507	FluB_HA	B/Hong Kong/CUHK18864/2011	Mar-2011
MF955508	FluB_HA	B/Hong Kong/CUHK18038/2011	Mar-2011
MF955509	FluB_HA	B/Hong Kong/CUHK62171/2008	Feb-2008
MF955510	FluB_HA	B/Hong Kong/CUHK42224/2010	Sep-2010
MF955511	FluB_HA	B/Hong Kong/CUHK39840/2010	Aug-2010
MF955512	FluB_HA	B/Hong Kong/CUHK39777/2012	Jul-2012
MF955513	FluB_HA	B/Hong Kong/CUHK32603/2012	Apr-2012
MF955514	FluB_HA	B/Hong Kong/CUHK27780/2011	Sep-2011
MF955515	FluB_HA	B/Hong Kong/CUHK22445/2011	May-2011
MF955516	FluB_HA	B/Hong Kong/CUHK24205/2011	Jul-2011
MF955517	FluB_HA	B/Hong Kong/CUHK51509/2002	Nov-2002
MF955518	FluB_HA	B/Hong Kong/CUHK6570/2003	Feb-2003
MF955519	FluB_HA	B/Hong Kong/CUHK35565/2002	Sep-2002
MF955520	FluB_HA	B/Hong Kong/CUHK13802/2001	Mar-2001
MF955521	FluB_HA	B/Hong Kong/CUHK653/1997	Feb-1997
MF955522	FluB_HA	B/Hong Kong/CUHK4029/1997	Mar-1997
MF955523	FluB_HA	B/Hong Kong/CUHK21776/1996	Jun-1996
MF955524	FluB_HA	B/Hong Kong/CUHK26643 /2012	Jan-2012
MF955525	FluB_HA	B/Hong Kong/CUHK10966/1996	Apr-1996
MF955526	FluB_HA	B/Hong Kong/CUHK4372/1997	Mar-1997
MF955527	FluB_HA	B/Hong Kong/CUHK11944/1999	Mar-1999
MF955528	FluB_HA	B/Hong Kong/CUHK11877 /1999	Mar-1999
MF955529	FluB_HA	B/Hong Kong/CUHK11930/1999	Mar-1999
MF955530	FluB_HA	B/Hong Kong/CUHK18313/1998	Sep-1998
MF955531	FluB_HA	B/Hong Kong/CUHK11073/1999	Feb-1999
MF955532	FluB_HA	B/Hong Kong/CUHK25187/2011	Jul-2011
MF955533	FluB_HA	B/Hong Kong/CUHK66139/2007	Apr-2007
MF955534	FluB_HA	B/Hong Kong/CUHK71746/2007	Jul-2007
MF955535	FluB_HA	B/Hong Kong/CUHK68911/2008	May-2008
MF955536	FluB_HA	B/Hong Kong/CUHK32249/2011	Dec-2011
MF955537	FluB_HA	B/Hong Kong/CUHK18142/2011	Mar-2011
MF955538	FluB_HA	B/Hong Kong/CUHK63456/2008	Mar-2008
MF955539	FluB_HA	B/Hong Kong/CUHK37722/2010	Jul-2010
MF955540	FluB_HA	B/Hong Kong/CUHK43052/2005	Jul-2005
MF955541	FluB_HA	B/Hong Kong/CUHK52158/2004	Aug-2004
MF955542	FluB_HA	B/Hong Kong/CUHK51212 /2004	Jul-2004
MF955543	FluB_HA	B/Hong Kong/CUHK53074/2006	Mar-2006
MF955544	FluB_HA	B/Hong Kong/CUHK52093/2004	Aug-2004
MF955545	FluB_HA	B/Hong Kong/CUHK21967/2000	Feb-2000
MF955546	FluB_HA	B/Hong Kong/CUHK30004/1999	Jun-1999
MF955547	FluB_HA	B/Hong Kong/CUHK20636/2000	Jan-2000
MF955548	FluB_HA	B/Hong Kong/CUHK13275/2002	Mar-2002
MF955549	FluB_HA	B/Hong Kong/CUHK10669/1996	Mar-1996
MF955550	FluB_HA	B/Hong Kong/CUHK4187/1997	Mar-1997
MF955551	FluB_HA	B/Hong Kong/CUHK4259/1997	Mar-1997
MF955552	FluB_HA	B/Hong Kong/CUHK4022/1997	Mar-1997
MF955553	FluB_HA	B/Hong Kong/CUHK740/1997	Feb-1997
MF955554	FluB_HA	B/Hong Kong/CUHK4008/1997	Mar-1997
MF955555	FluB_HA	B/Hong Kong/CUHK18502/1998	Sep-1998
MF955556	FluB_HA	B/Hong Kong/CUHK14952 /1999	Jun-1999
MF955557	FluB_HA	B/Hong Kong/CUHK30046 /1999	Jun-1999
MF955558	FluB_HA	B/Hong Kong/CUHK10906/1999	Feb-1999
MF955559	FluB_HA	B/Hong Kong/CUHK11988 /1999	Mar-1999
MF955560	FluB_HA	B/Hong Kong/CUHK10724/1996	Mar-1996
MF955561	FluB_HA	B/Hong Kong/CUHK40333/2010	Sep-2010
MF955562	FluB_HA	B/Hong Kong/CUHK13314 /2011	Jan-2011
MF955563	FluB_HA	B/Hong Kong/CUHK44277/2010	Nov-2010
MF955564	FluB_HA	B/Hong Kong/CUHK16218/2011	Feb-2011
MF955565	FluB_HA	B/Hong Kong/CUHK27583/2012	Feb-2012
MF955566	FluB_HA	B/Hong Kong/CUHK10701/1996	Mar-1996
MF955567	FluB_HA	B/Hong Kong/CUHK26627/2012	Jan-2012
MF955568	FluB_HA	B/Hong Kong/CUHK22381/2011	May-2011
MF955569	FluB_HA	B/Hong Kong/CUHK24779 /2011	Jul-2011
MF955570	FluB_HA	B/Hong Kong/CUHK22332/2003	Mar-2003

GenBank accession no.	Type	Isolate ID	Collection date
MF955571	FluB_HA	B/Hong Kong/CUHK51098/2004	Jul-2004
MF955572	FluB_HA	B/Hong Kong/CUHK57325/2006	May-2006
MF955573	FluB_HA	B/Hong Kong/CUHK22674/2001	May-2001
MF955574	FluB_HA	B/Hong Kong/CUHK5815/2001	Jan-2001
MF955575	FluB_HA	B/Hong Kong/CUHK22878/2002	May-2002
MF955576	FluB_HA	B/Hong Kong/CUHK6649/2002	Feb-2002
MF955577	FluB_HA	B/Hong Kong/CUHK5696/2001	Jan-2001
MF955578	FluB_HA	B/Hong Kong/CUHK6335/2002	Feb-2002
MF955579	FluB_HA	B/Hong Kong/CUHK23689/2000	Apr-2000
MF955580	FluB_HA	B/Hong Kong/CUHK27366/2000	Aug-2000
MF955581	FluB_HA	B/Hong Kong/CUHK11665/1999	Feb-1999
MF955582	FluB_HA	B/Hong Kong/CUHK14563/1999	Jun-1999
MF955583	FluB_HA	B/Hong Kong/CUHK13246/2002	Mar-2002
MF955584	FluB_HA	B/Hong Kong/CUHK23440/2002	May-2002
MF955585	FluB_HA	B/Hong Kong/CUHK21724/2002	Apr-2002
MF955586	FluB_HA	B/Hong Kong/CUHK22825/2001	May-2001
MF955587	FluB_HA	B/Hong Kong/CUHK6935/2002	Feb-2002
MF955588	FluB_HA	B/Hong Kong/CUHK18145/1998	Sep-1998
MF955589	FluB_HA	B/Hong Kong/CUHK10522/1996	Mar-1996
MF955590	FluB_HA	B/Hong Kong/CUHK10806/1996	Apr-1996
MF955591	FluB_HA	B/Hong Kong/CUHK3469/1996	Mar-1996
MF955592	FluB_HA	B/Hong Kong/CUHK2829/1996	Jan-1996
MF955593	FluB_HA	B/Hong Kong/CUHK3445/1996	Mar-1996
MF955594	FluB_HA	B/Hong Kong/CUHK721/1997	Feb-1997
MF955595	FluB_HA	B/Hong Kong/CUHK18654/1998	Sep-1998
MF955596	FluB_HA	B/Hong Kong/CUHK15890/1998	Jun-1998
MF955597	FluB_HA	B/Hong Kong/CUHK17459/1998	Aug-1998
MF955598	FluB_HA	B/Hong Kong/CUHK4261/1997	Mar-1997
MF955599	FluB_HA	B/Hong Kong/CUHK4304/1997	Mar-1997
MF955600	FluB_HA	B/Hong Kong/CUHK52533/2004	Aug-2004
MF955601	FluB_HA	B/Hong Kong/CUHK52708/2004	Aug-2004
MF955602	FluB_HA	B/Hong Kong/CUHK52437/2004	Aug-2004
MF955603	FluB_HA	B/Hong Kong/CUHK52248 /2006	Feb-2006
MF955604	FluB_HA	B/Hong Kong/CUHK68586/2007	May-2007
MF955605	FluB_HA	B/Hong Kong/CUHK67604/2007	May-2007
MF955606	FluB_HA	B/Hong Kong/CUHK50698 /2006	Jan-2006
MF955607	FluB_HA	B/Hong Kong/CUHK52419/2004	Aug-2004
MF955608	FluB_HA	B/Hong Kong/CUHK34606/2002	Aug-2002
MF955609	FluB_HA	B/Hong Kong/CUHK24152/2002	Jun-2002
MF955610	FluB_HA	B/Hong Kong/CUHK6521/2003	Feb-2003
MF955611	FluB_HA	B/Hong Kong/CUHK13268/2003	Feb-2003
MF955612	FluB_HA	B/Hong Kong/CUHK5672/2003	Jan-2003
MF955613	FluB_HA	B/Hong Kong/CUHK6681/2003	Feb-2003
MF955614	FluB_HA	B/Hong Kong/CUHK22432/2002	Apr-2002
MF955615	FluB_HA	B/Hong Kong/CUHK52912/2000	Dec-2000
MF955616	FluB_HA	B/Hong Kong/CUHK8835/2005	Mar-2005
MF955617	FluB_HA	B/Hong Kong/CUHK52684/2004	Aug-2004
MF955618	FluB_HA	B/Hong Kong/CUHK52196 /2004	Aug-2004
MF955619	FluB_HA	B/Hong Kong/CUHK23890 /2000	Apr-2000
MF955620	FluB_HA	B/Hong Kong/CUHK16057/1998	Jun-1998
MF955621	FluB_HA	B/Hong Kong/CUHK16801/1998	Jul-1998
MF955622	FluB_HA	B/Hong Kong/CUHK4232/1997	Mar-1997
MF955623	FluB_HA	B/Hong Kong/CUHK532/1997	Jan-1997
MF955624	FluB_HA	B/Hong Kong/CUHK939/1997	Feb-1997
MF955625	FluB_HA	B/Hong Kong/CUHK17447/1998	Aug-1998
MF955626	FluB_HA	B/Hong Kong/CUHK52212/2000	Dec-2000
MF955627	FluB_HA	B/Hong Kong/CUHK20580/2000	Jan-2000
MF955628	FluB_HA	B/Hong Kong/CUHK14181 /1999	May-1999
MF955629	FluB_HA	B/Hong Kong/CUHK18025 /1998	Aug-1998
MF955630	FluB_HA	B/Hong Kong/CUHK14449/1999	Jun-1999
MF955631	FluB_HA	B/Hong Kong/CUHK29311/2011	Oct-2011
MF955632	FluB_HA	B/Hong Kong/CUHK33217/2011	Dec-2011
MF955633	FluB_HA	B/Hong Kong/CUHK28674/2011	Sep-2011
MF955634	FluB_HA	B/Hong Kong/CUHK16069/2011	Feb-2011
MF955635	FluB_HA	B/Hong Kong/CUHK25561/2011	Jul-2011
MF955636	FluB_HA	B/Hong Kong/CUHK30018/2012	Mar-2012
MF955637	FluB_HA	B/Hong Kong/CUHK3472/1996	Mar-1996
MF955638	FluB_HA	B/Hong Kong/CUHK10610/1996	Mar-1996
MF955639	FluB_HA	B/Hong Kong/CUHK3331/1996	Mar-1996

GenBank accession no.	Type	Isolate ID	Collection date
MF955640	FluB_HA	B/Hong Kong/CUHK30694/2012	Mar-2012
MF955641	FluB_HA	B/Hong Kong/CUHK34334/2012	May-2012
MF955642	FluB_HA	B/Hong Kong/CUHK44360/2007	Oct-2007
MF955643	FluB_HA	B/Hong Kong/CUHK29953/2010	Mar-2010
MF955644	FluB_HA	B/Hong Kong/CUHK61550/2007	Jan-2007
MF955645	FluB_HA	B/Hong Kong/CUHK31105/2011	Nov-2011
MF955646	FluB_HA	B/Hong Kong/CUHK67267/2012	Jan-2012
MF955647	FluB_HA	B/Hong Kong/CUHK27078/2011	Aug-2011
MF955648	FluB_HA	B/Hong Kong/CUHK39059/2010	Aug-2010
MF955649	FluB_HA	B/Hong Kong/CUHK36243/2012	May-2012
MF955650	FluB_HA	B/Hong Kong/CUHK29160/1996	Aug-1996
MF955651	FluB_HA	B/Hong Kong/CUHK16792/1998	Jul-1998
MF955652	FluB_HA	B/Hong Kong/CUHK28577/1996	Aug-1996
MF955653	FluB_HA	B/Hong Kong/CUHK48264/2012	Dec-2012
MF955654	FluB_HA	B/Hong Kong/CUHK30062/2012	Mar-2012
MF955655	FluB_HA	B/Hong Kong/CUHK37079/2010	Jun-2010
MF955656	FluB_HA	B/Hong Kong/CUHK71157/2007	Jul-2007
MF955657	FluB_HA	B/Hong Kong/CUHK47768/2007	Dec-2007
MF955658	FluB_HA	B/Hong Kong/CUHK72892/2007	Jul-2007
MF955659	FluB_HA	B/Hong Kong/CUHK64525/2007	Mar-2007
MF955660	FluB_HA	B/Hong Kong/CUHK68325/2007	May-2007
MF955661	FluB_HA	B/Hong Kong/CUHK35400/2010	May-2010
MF955662	FluB_HA	B/Hong Kong/CUHK67554/2008	Apr-2008
MF955663	FluB_HA	B/Hong Kong/CUHK67100 /2008	Apr-2008
MF955664	FluB_HA	B/Hong Kong/CUHK69311/2007	Jun-2007
MF955665	FluB_HA	B/Hong Kong/CUHK57526/2006	May-2006
MF955666	FluB_HA	B/Hong Kong/CUHK8074/2005	Mar-2005
MF955667	FluB_HA	B/Hong Kong/CUHK30407/2011	Oct-2011
MF955668	FluB_HA	B/Hong Kong/CUHK26732/2012	Jan-2012
MF955669	FluB_HA	B/Hong Kong/CUHK29792/2011	Oct-2011
MF955670	FluB_HA	B/Hong Kong/CUHK15388/2011	Feb-2011
MF955671	FluB_HA	B/Hong Kong/CUHK18618/1998	Sep-1998
MF955672	FluB_HA	B/Hong Kong/CUHK11469 /1999	Feb-1999
MF955673	FluB_HA	B/Hong Kong/CUHK51669/2004	Jul-2004
MF955674	FluB_HA	B/Hong Kong/CUHK11794 /1999	Mar-1999
MF955675	FluB_HA	B/Hong Kong/CUHK22891/2002	May-2002
MF955676	FluB_HA	B/Hong Kong/CUHK34393/2002	Aug-2002
JX429604	FluB_HA	B/Hong Kong/CUHK22975/2001	May-2001
JX429608	FluB_HA	B/Hong Kong/CUHK34987/2002	Aug-2002
JX429609	FluB_HA	B/Hong Kong/CUHK35884/2002	Sep-2002
JX429614	FluB_HA	B/Hong Kong/CUHK5597/2003	Jan-2003
JX429615	FluB_HA	B/Hong Kong/CUHK5879/2003	Jan-2003
JX429617	FluB_HA	B/Hong Kong/CUHK6081/2003	Jan-2003
JX429618	FluB_HA	B/Hong Kong/CUHK6088/2003	Jan-2003
JX429619	FluB_HA	B/Hong Kong/CUHK6212/2003	Feb-2003
JX429620	FluB_HA	B/Hong Kong/CUHK6320/2003	Feb-2003
JX429621	FluB_HA	B/Hong Kong/CUHK6327/2003	Feb-2003
JX429622	FluB_HA	B/Hong Kong/CUHK6328/2003	Feb-2003
JX429623	FluB_HA	B/Hong Kong/CUHK6343/2003	Feb-2003
JX429624	FluB_HA	B/Hong Kong/CUHK6550/2003	Feb-2003
JX429625	FluB_HA	B/Hong Kong/CUHK22269/2003	Mar-2003
JX429626	FluB_HA	B/Hong Kong/CUHK58582/2004	Dec-2004
JX429627	FluB_HA	B/Hong Kong/CUHK5196/2005	Jan-2005
JX429628	FluB_HA	B/Hong Kong/CUHK6241/2005	Jan-2005
JX429629	FluB_HA	B/Hong Kong/CUHK6331/2005	Jan-2005
JX429630	FluB_HA	B/Hong Kong/CUHK12098/2005	May-2005
JX429631	FluB_HA	B/Hong Kong/CUHK44257/2005	Aug-2005
JX429632	FluB_HA	B/Hong Kong/CUHK46869/2005	Sep-2005
JX429633	FluB_HA	B/Hong Kong/CUHK49537/2005	Nov-2005
JX429634	FluB_HA	B/Hong Kong/CUHK50098 /2006	Jan-2006
JX429635	FluB_HA	B/Hong Kong/CUHK50543/2006	Jan-2006
JX429637	FluB_HA	B/Hong Kong/CUHK51122/2006	Jan-2006
JX429638	FluB_HA	B/Hong Kong/CUHK51422/2006	Feb-2006
JX429639	FluB_HA	B/Hong Kong/CUHK55362/2006	Apr-2006
JX429640	FluB_HA	B/Hong Kong/CUHK55544/2006	Apr-2006
JX429641	FluB_HA	B/Hong Kong/CUHK56028 /2006	Apr-2006
JX429642	FluB_HA	B/Hong Kong/CUHK56533/2006	May-2006
JX429643	FluB_HA	B/Hong Kong/CUHK56837/2006	May-2006
JX429644	FluB_HA	B/Hong Kong/CUHK57008/2006	May-2006

GenBank accession no.	Type	Isolate ID	Collection date
JX429645	FluB_HA	B/Hong Kong/CUHK63364/2006	Aug-2006
JX429646	FluB_HA	B/Hong Kong/CUHK65660/2006	Oct-2006
JX429647	FluB_HA	B/Hong Kong/CUHK66461 /2006	Oct-2006
JX429648	FluB_HA	B/Hong Kong/CUHK48904 /2007	Dec-2007
JX429649	FluB_HA	B/Hong Kong/CUHK60082/2008	Jan-2008
JX429650	FluB_HA	B/Hong Kong/CUHK63116/2005	Dec-2005
JX429651	FluB_HA	B/Hong Kong/CUHK64327 /2007	Mar-2007
JX429653	FluB_HA	B/Hong Kong/CUHK67032/2008	Apr-2008
JX429654	FluB_HA	B/Hong Kong/CUHK25867/2010	Jan-2010
JX429656	FluB_HA	B/Hong Kong/CUHK28755/2010	Feb-2010
JX429660	FluB_HA	B/Hong Kong/CUHK30950/2010	Mar-2010
JX429666	FluB_HA	B/Hong Kong/CUHK6400/2003	Feb-2003
JX429667	FluB_HA	B/Hong Kong/CUHK45094/2005	Aug-2005
JX429668	FluB_HA	B/Hong Kong/CUHK26861/2010	Jan-2010
JX429673	FluB_HA	B/Hong Kong/CUHK23362/2000	Apr-2000
JX429674	FluB_HA	B/Hong Kong/CUHK24511/2000	May-2000
JX429676	FluB_HA	B/Hong Kong/CUHK26708/2000	Jul-2000
JX429677	FluB_HA	B/Hong Kong/CUHK28151/2000	Sep-2000
JX429679	FluB_HA	B/Hong Kong/CUHK45490/2000	Nov-2000
JX429682	FluB_HA	B/Hong Kong/CUHK45810 /2000	Nov-2000
JX429683	FluB_HA	B/Hong Kong/CUHK52855/2000	Dec-2000
JX429684	FluB_HA	B/Hong Kong/CUHK5140/2001	Jan-2001
JX429685	FluB_HA	B/Hong Kong/CUHK5678/2001	Jan-2001
JX429686	FluB_HA	B/Hong Kong/CUHK5792/2001	Jan-2001
JX429687	FluB_HA	B/Hong Kong/CUHK5813/2001	Jan-2001
JX429689	FluB_HA	B/Hong Kong/CUHK5949/2001	Jan-2001
JX429690	FluB_HA	B/Hong Kong/CUHK13723/2001	Mar-2001
JX429691	FluB_HA	B/Hong Kong/CUHK13803/2001	Mar-2001
JX429692	FluB_HA	B/Hong Kong/CUHK22328/2001	May-2001
JX429693	FluB_HA	B/Hong Kong/CUHK22361/2001	May-2001
JX429694	FluB_HA	B/Hong Kong/CUHK22394/2001	May-2001
JX429697	FluB_HA	B/Hong Kong/CUHK22731/2001	May-2001
JX429698	FluB_HA	B/Hong Kong/CUHK22941/2001	May-2001
JX429699	FluB_HA	B/Hong Kong/CUHK22980/2001	May-2001
JX429702	FluB_HA	B/Hong Kong/CUHK34740/2002	Aug-2002
JX429703	FluB_HA	B/Hong Kong/CUHK35386/2002	Sep-2002
JX429706	FluB_HA	B/Hong Kong/CUHK50602/2002	Oct-2002
JX429709	FluB_HA	B/Hong Kong/CUHK52162/2002	Nov-2002
JX429710	FluB_HA	B/Hong Kong/CUHK5107/2003	Jan-2003
JX429712	FluB_HA	B/Hong Kong/CUHK21937/2003	Mar-2003
JX429713	FluB_HA	B/Hong Kong/CUHK51379/2003	Jul-2003
JX429714	FluB_HA	B/Hong Kong/CUHK71975/2003	Sep-2003
JX429715	FluB_HA	B/Hong Kong/CUHK73990/2003	Nov-2003
JX429716	FluB_HA	B/Hong Kong/CUHK33398/2004	Apr-2004
JX429717	FluB_HA	B/Hong Kong/CUHK33528/2004	May-2004
JX429721	FluB_HA	B/Hong Kong/CUHK50385/2004	Jul-2004
JX429722	FluB_HA	B/Hong Kong/CUHK50636/2004	Jul-2004
JX429724	FluB_HA	B/Hong Kong/CUHK51106/2004	Jul-2004
JX429725	FluB_HA	B/Hong Kong/CUHK51149 /2004	Jul-2004
JX429726	FluB_HA	B/Hong Kong/CUHK52216/2004	Aug-2004
JX429727	FluB_HA	B/Hong Kong/CUHK52640/2004	Aug-2004
JX429728	FluB_HA	B/Hong Kong/CUHK52705/2004	Aug-2004
JX429729	FluB_HA	B/Hong Kong/CUHK53722/2004	Sep-2004
JX429731	FluB_HA	B/Hong Kong/CUHK5891 /2005	Jan-2005
JX429732	FluB_HA	B/Hong Kong/CUHK6337/2005	Jan-2005
JX429733	FluB_HA	B/Hong Kong/CUHK6702/2005	Feb-2005
JX429734	FluB_HA	B/Hong Kong/CUHK7123/2005	Feb-2005
JX429735	FluB_HA	B/Hong Kong/CUHK7597/2005	Feb-2005
JX429737	FluB_HA	B/Hong Kong/CUHK8338/2005	Mar-2005
JX429738	FluB_HA	B/Hong Kong/CUHK8842/2005	Mar-2005
JX429739	FluB_HA	B/Hong Kong/CUHK11134/2005	Apr-2005
JX429740	FluB_HA	B/Hong Kong/CUHK43516/2007	Sep-2007
JX429741	FluB_HA	B/Hong Kong/CUHK44142/2007	Sep-2007
JX429742	FluB_HA	B/Hong Kong/CUHK44997/2007	Oct-2007
JX429743	FluB_HA	B/Hong Kong/CUHK47361/2005	Oct-2005
JX429745	FluB_HA	B/Hong Kong/CUHK66862/2006	Oct-2006
JX429746	FluB_HA	B/Hong Kong/CUHK40165/2005	Jun-2005
JX429748	FluB_HA	B/Hong Kong/CUHK67089/2007	Apr-2007
JX429749	FluB_HA	B/Hong Kong/CUHK63087/2007	Feb-2007

GenBank accession no.	Type	Isolate ID	Collection date
JX429751	FluB_HA	B/Hong Kong/CUHK68567/2007	May-2007
JX429752	FluB_HA	B/Hong Kong/CUHK74092/2007	Aug-2007
JX429753	FluB_HA	B/Hong Kong/CUHK74822/2007	Aug-2007
JX429754	FluB_HA	B/Hong Kong/CUHK61247/2008	Jan-2008
JX429756	FluB_HA	B/Hong Kong/CUHK63126/2008	Feb-2008
JX429757	FluB_HA	B/Hong Kong/CUHK63367/2008	Mar-2008
JX429758	FluB_HA	B/Hong Kong/CUHK63832 /2008	Mar-2008
JX429760	FluB_HA	B/Hong Kong/CUHK64054/2008	Mar-2008
JX429763	FluB_HA	B/Hong Kong/CUHK69306/2008	May-2008
JX429764	FluB_HA	B/Hong Kong/CUHK69850/2008	May-2008
JX429766	FluB_HA	B/Hong Kong/CUHK28267/2010	Feb-2010
JX429767	FluB_HA	B/Hong Kong/CUHK30805/2010	Mar-2010
JX429769	FluB_HA	B/Hong Kong/CUHK32800/2010	Apr-2010
JX429770	FluB_HA	B/Hong Kong/CUHK34437/2010	May-2010
JX429771	FluB_HA	B/Hong Kong/CUHK38197/2010	Jul-2010
JX429776	FluB_HA	B/Hong Kong/CUHK21905 /2000	Feb-2000
JX429778	FluB_HA	B/Hong Kong/CUHK21910/2000	Feb-2000
JX429779	FluB_HA	B/Hong Kong/CUHK6017/2001	Feb-2001
JX429780	FluB_HA	B/Hong Kong/CUHK13773/2001	Mar-2001
JX429781	FluB_HA	B/Hong Kong/CUHK13865/2001	Mar-2001
JX429782	FluB_HA	B/Hong Kong/CUHK70022/2003	Sep-2003
JX429785	FluB_HA	B/Hong Kong/CUHK50947 /2004	Jul-2004
JX429786	FluB_HA	B/Hong Kong/CUHK63150/2008	Feb-2008
JX429787	FluB_HA	B/Hong Kong/CUHK63194/2008	Feb-2008
JX429788	FluB_HA	B/Hong Kong/CUHK81112/2008	Dec-2008
JX429790	FluB_HA	B/Hong Kong/CUHK54261/2006	Mar-2006
JX429791	FluB_HA	B/Hong Kong/CUHK51357/2004	Jul-2004

*A total of 437 influenza A and 396 influenza B isolates were collected.

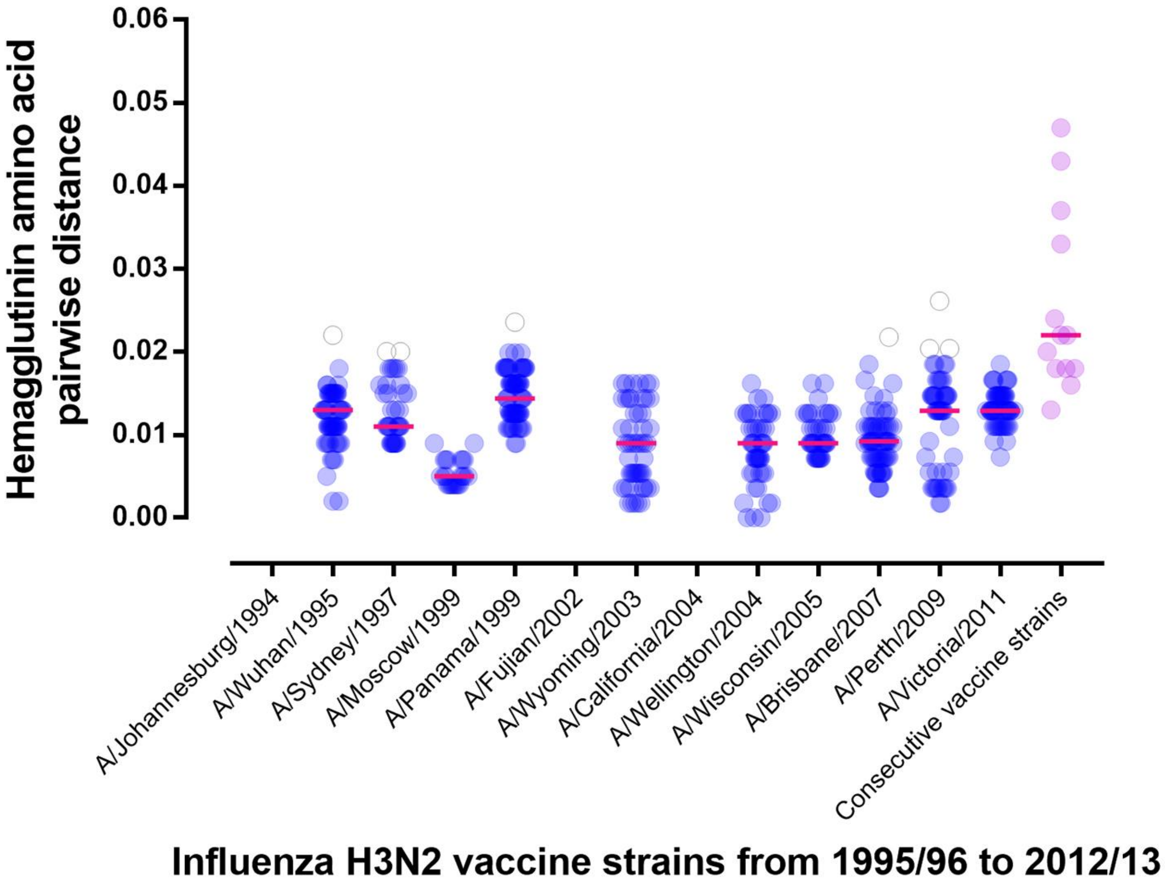

Technical Appendix Figure 1. Pairwise hemagglutinin amino acid distances between circulating and vaccine strains of influenza A(H3N2), Hong Kong, China, 1996–2012. The study analyzed 502 circulating A(H3N2) viruses. Calculation was performed using a Poisson correction model with rate variation among sites assumed in gamma distribution. Alignment positions containing gaps were masked. All analyses were performed in MEGA 6 (https://www.megasoftware.net/). Red horizontal lines denote median values. Outliers (distance >2 SD) are shown as empty circles. The mean distance between circulating viruses and vaccine strains ranged from 0.009 to 0.014, indicating close relationship. All vaccine strains were recommended by the World Health Organization for both Northern and Southern Hemispheres, except A/Wellington/2004, which was recommended for Southern Hemisphere vaccines only. The mean distance between consecutive vaccine strains was 0.02.

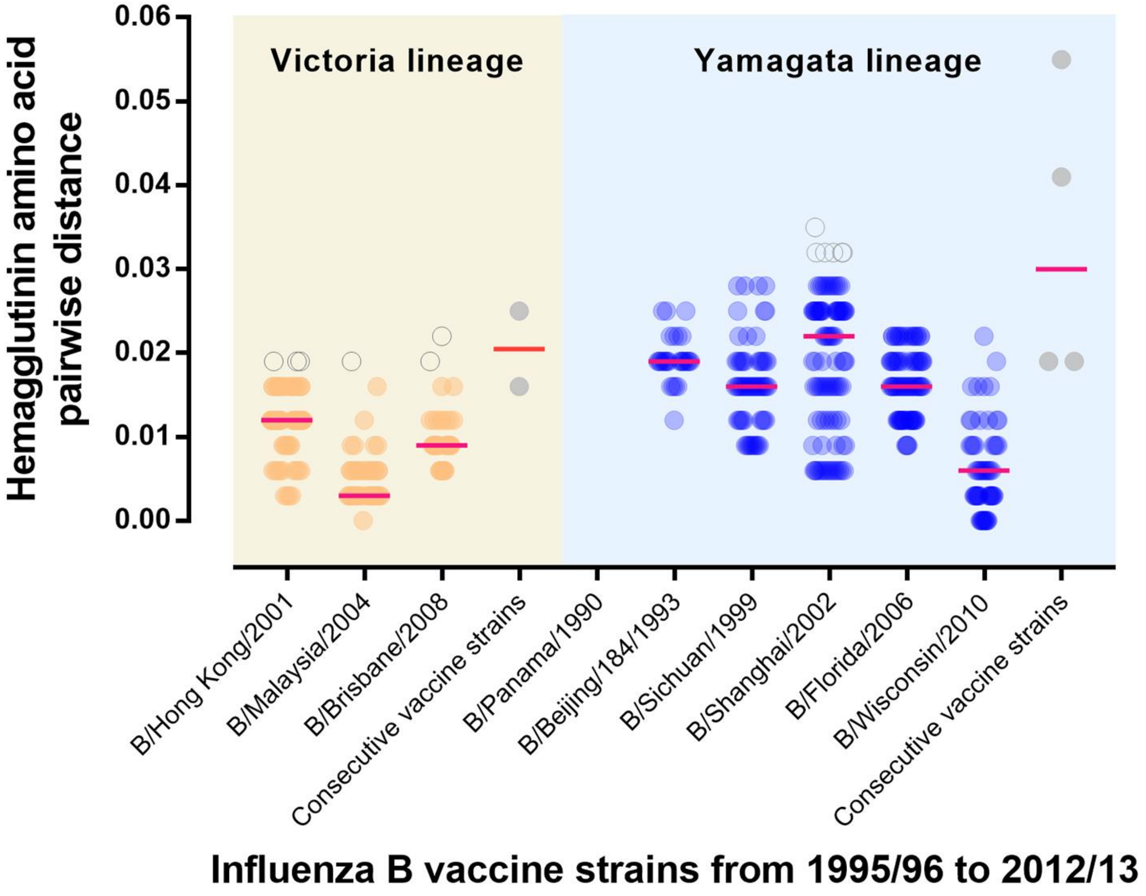

Technical Appendix Figure 2. Pairwise hemagglutinin amino acid distance between circulating and vaccine strains of influenza B, Hong Kong, China, 1996–2012. The study analyzed 481 circulating influenza B viruses. Calculation was performed using a Poisson correction model with rate variation among sites assumed in gamma distribution. Alignment positions containing gaps were masked. All analyses were performed in MEGA 6 (https://www.megasoftware.net/). Red horizontal lines denote median values. Outliers (distance >2 SD deviations) are shown as empty circles. The mean distance between circulating viruses and vaccine strains ranged from 0.003 to 0.012 and from 0.006 to 0.019 for the Victoria and Yamagata lineages, respectively. The mean distance between consecutive vaccine strains was 0.02 and 0.03 for Victoria and Yamagata lineages, respectively.
